# Phenomenology of Neapolitan Pizza Baking in a Traditional Wood-Fired Oven

**DOI:** 10.3390/foods12040890

**Published:** 2023-02-19

**Authors:** Aniello Falciano, Mauro Moresi, Paolo Masi

**Affiliations:** 1 Department of Agricultural Sciences, University of Naples Federico II, 80055 Portici, Italy; 2 Academic Centre for Food Innovation, University of Naples Federico II, 80055 Portici, Italy; 3 Department for Innovation in the Biological Agrofood and Forestry Systems, University of Tuscia, 01100 Viterbo, Italy

**Keywords:** baking characterization, browning and burning kinetics, infrared thermal scanning, Neapolitan pizza, raised rim growth, thermal mapping of pizza crust and bottom, visual color assessment, weight loss, wood-fired oven

## Abstract

Despite Neapolitan pizza is a globally renowned Italian food, its obligatory baking in wood-fired ovens has so far received little attention in the scientific community. Since heat transfer during pizza baking is not at all uniform, the main aim of this work was to analyze the phenomenology of Neapolitan pizza baking in a pilot-scale wood-fired pizza oven operating in quasi steady-state conditions. The different upper area sections of pizza covered or not by the main topping ingredients (i.e., tomato puree, sunflower oil, or mozzarella cheese), as well the bottom of the pizza and the growth of its raised rim, were characterized by visual colorimetric analysis, while the time course of their corresponding temperatures was monitored using an infrared thermal scanning camera. The maximum temperature of the pizza bottom was equal to 100 ± 9 °C, while that of the upper pizza side ranged from 182 °C to 84 or 67 °C in the case of white pizza, tomato pizza, or margherita pizza, respectively, mainly because of their diverse moisture content and emissivity. The pizza weight loss was nonlinearly related to the average temperature of the upper pizza side. The formation of brown or black colored areas on the upper and lower sides of baked pizza was detected with the help of an electronic eye. The upper side exhibited greater degrees of browning and blackening than the lower one, their maximum values being about 26 and 8%, respectively, for white pizza. These results might help develop a specific modelling and monitoring strategy to reduce variability and maximize the quality attributes of Neapolitan pizza.

## 1. Introduction

The Neapolitan pizza is part of the traditional specialties guaranteed (TSG) and has to be baked in wood-fired ovens [1]. Its final quality strictly depends on the ability of the Neapolitan pizza maker (*pizzaiuolo*), his/her art having been included on the List of Intangible Cultural Heritage of Humanity [2].

Even if the pizza production stages from dough preparation to baking have been thoroughly illustrated [3], how wood-fired pizza ovens should be appropriately operated to assure a soft, elastic, tender, and fragrant Neapolitan-style wood-fired pizza with a crust finely bubbled up and just charred in a few spots is one of the pizzaiuolo skills patiently learned after long apprenticeships. The charring is a byproduct of baking the pizza in a blazing-hot oven. It mainly affects the raised edge and underside areas of the crust, which are nearest to the oven heat sources (oven vault and floor, respectively). It would result in burning if the pizza were baked any longer than the recommended 90 s [1].

The formation of color in pizza during baking is generally expressed as browning and is the result of non-enzymatic chemical reactions, such as the Maillard reaction and caramelization. Under direct heating, the former occurs between reducing sugars and amino acids, proteins, and/or other nitrogenous organic compounds, while the latter between carbohydrates, mainly sucrose and reducing sugars [4]. Both reactions only depend on temperature and water activity, this expressing the readiness of water for chemical reactions in food products. Among the numerous methods used to quantify the kinetics of browning via color measurements and chemical analysis, visual color change of food products has been successfully described using the CIE-Lab color indices [5,6,7].

During the pizza baking process in a wood-fired oven, simultaneous heat and mass transfer takes place within the product inducing a number of physical, chemical, and biochemical changes in addition to browning, such as volume expansion and shrinkage, water evaporation, dough/crumb transition owing to protein denaturation and starch gelatinization, and the formation of a crust [3,8]. The operation of a pilot-scale wood-fired pizza oven from its start-up phase to its operation in quasi steady-state conditions was previously described [9]. Moreover, it was assessed that its average thermal efficiency was 13 ± 4% when baking both white and tomato pizza products. Then, Falciano et al. [10] succeeded in quantifying that the heat loss rates through flue gas and the insulated oven chamber were, respectively, equal to 46% and 26% of the energy supplied by burning firewood, while the heat accumulation rate in the firebrick oven was near to 3.4 kW. This was sufficient not only to maintain the temperatures of the oven vault and floor practically constant at (546 ± 53) °C and (453 ± 32) °C, respectively, but also to bake one or two pizzas at the same time [9]. Such heat flow rate was predicted by accounting for the simultaneous heat transfer mechanisms of radiation and convection between the oven vault and floor surface areas. Moreover, a series of water heating tests were quite accurately reconstructed by accounting for a simultaneous heat flow from the oven vault of the radiative and convective types and from the oven floor of the conductive one, their contribution representing about 73%, 15%, and 12% of the overall heat transferred, respectively.

The main aim of this work was to characterize the phenomenology of Neapolitan pizza baking in a pilot-scale wood-fired oven operating in quasi steady-state conditions. Since heat transfer during pizza baking is not at all uniform, and particularly complex, the temperature of the upper central area of the pizza, being covered by diverse topping ingredients differing in their thermal properties, exhibits a slower rise than that of the external annular rim, this being devoid of any topping. The rim undergoes a greater expansion due to the rapid evaporation of its water content. As the temperature continues to increase, gluten proteins experience aggregation and cross-linking, this conferring rigidity to the alveolar structure formed that does not collapse but becomes permanent. Any further increase in the temperature of the raised rim, as well as in that of the lower side of pizza laid upon the hot oven floor, causes a strong reduction in the moisture content and triggers pyrolysis reactions with the formation of diffuse burns. Thus, the first aim of this work was to measure the different area sections of pizza covered or not by the main topping ingredients (i.e., tomato puree, sunflower oil, or mozzarella cheese), as well as the growth of the raised rim, by image analysis. The second and third aims were to monitor the time course of the temperature of the aforementioned areas and of the pizza weight loss during the baking of pizza samples differently garnished. The final one was to monitor the evolution of the degree of browning or burning of the pizza samples undergoing baking by means of an electronic eye and develop a kinetic model able to describe the extent of browned and blackened areas as a function of time and temperature.

## 2. Materials and Methods

### 2.1. Raw Materials

The Neapolitan pizza bases were prepared using the following ingredients: (i) soft wheat flour type 00 with 12% (*w*/*w*) nominal moisture content (Antimo Caputo Srl, Naples, Italy); (ii) fresh brewer’s yeast (Lesaffre Italia, Trecasali, Parma, Italy); (iii) Sicilian fine table salt (Italkali, Petralia, Palermo, Italy); and (iv) deionized water at 16–18 °C. Each pizza base was baked as such or garnished using sunflower oil (Mepa Srl, Terzigno, Naples, Italy) and/or tomato puree at 7.0 ± 0.2° Brix (Mutti SpA, Parma, Italy), and Mozzarella cheese (Selex Gruppo Commerciale SpA, Milan, Italy). The latter had a moisture content of 50% *w*/*w* on a wet basis. Seasoned oak logs with weight, length, and diameter equal to 600 ± 200 g, 250 ± 20 mm, and 40 ± 10 mm, respectively, were used as firewood.

### 2.2. Pizza Preparation

The pizza dough was prepared, leavened, and manually laminated [9] by a professional pizza maker (i.e., Mr. Enzo Coccia, Pizzeria La Notizia, Naples, Italy) to assure data reproducibility. The final pizza shell was finally baked as such (sample A) or topped as shown in Table 1 (samples B–E).

### 2.3. Equipment

The pilot-scale wood-fired pizza oven used in this work is shown in Figure S1 in the electronic supplement. Its geometry and start-up procedure were previously described [9].

### 2.4. Baking Tests

All tests were carried out in triplicate after the oven had reached quasi steady-state operating conditions, this requiring an oak log feed rate (Q_fw_) of 3 kg/h for not less than 6 h [9]. Each pizza sample of the 5 types shown in Table 1 was then baked for 20, 40, 60, 80, or 100 s. As soon as each pizza had been removed from the oven, the temperature of the oven floor area previously occupied by the sample itself, as well as that of the annular area around such a sample, was measured by using an infra-red (IR) thermal imaging camera (FLIR E95 42°, FLIR System OU, Tallinn, Estonia). As soon as the pizza sample had been extracted from the oven, the temperatures of the pizza shell in the rim, and upper and lower central areas were measured using the above thermal imaging camera. Finally, the sample was weighted using an analytical balance (Gibertini, Milan, Italy) to assess its weight loss.

### 2.5. Monitoring of the Raised Rim Height

The variation in the instantaneous height (h) of the raised rim during the baking phase was assessed by using a thermal imaging camera (FLIR E95 42°, FLIR System OU, Tallinn, Estonia) operating in the video mode, which had been fixed on a stand, while a metal reference ruler was positioned near to the pizza sample inside the oven. The images of the pizza sample were extrapolated from the registered video for an overall baking time (t_B_) of 80 s. The images were captured every 2 s during the first 20 s, every 4 s as t_B_ ranged from 20 to 40 s, and finally every 10 s as t_B_ increased from 40 to 80 s. These were then analyzed using a free, open-source image processing software ImageJ (Java2HTML v. 1.5, National Institutes of Health, Bethesda, MD, USA).

### 2.6. Color Visual Assessment of Baked Pizza Areas

The variation in the color of each pizza sample undergoing baking in a wood-fired oven was monitored using the IRIS visual analyzer 400 and AlphaSoft software (Alpha MOS, Toulouse, France). The pictures of each pizza sample were taken in a closable light chamber (420 × 560 × 380 mm) to assure controlled light conditions and avoid any influence of external light on the visual analysis. A dual top and bottom LED (light emitting diodes) lighting system was used to prevent any shadow effect. It was characterized by a color temperature of 6700 K, a color rendering index (CRI) of 98 (this involving an excellent ability of the light source to accurately reproduce the colors of the object it illuminates, its maximum score being equal to 100), and spectral power distribution of natural daylight close to D65 corresponding to the color temperature of the sky on a clear day around noon. The acA2500-14gc Basler ace GigE camera (Basler AG, Ahrensburg, Germany) equipped with 16-mm diameter lens was used to shoot the pizza sample pictures. Once the instrument had been calibrated with a certified color scale, the pizza samples were placed over a removable white tray, this diffusing a uniform light inside the aforementioned light chamber. Measurements on both the upper and lower pizza sides were performed in triplicate using the CIELab color space, this being an international standard for color measurement [11]. L* describes brightness and extends from 0 (black) to 100 (white), while a* and b* represent the green vs. red, and blue vs. yellow coordinates, each one ranging from −100 to +100. In accordance with Sadowska et al. [12], once the background of each picture had been removed, the edited image was processed as a color spectrum representing the surface area percentage occupied by each color identified on the pizza surface within a fixed scale of 4096 colors. Each of these colors corresponds to a unique set of 3 values in the RGB (R—red, G—green, B—blue) color space (see the online calculator at https://www.checkyourmath.com/convert/color/decimal_rgb.php, accessed on 13 February 2023). These coordinates describe the relative amounts of red, green, and blue light mixed to create a particular color, each one ranging from 0 (no color added) to 255 (100% color added). The values for parameters R, G, and B were averaged and accounted for the frequency of appearance of each individual color decimal code. The hierarchical cluster analysis (HCA) was used to create clusters of colors corresponding to the degree of browning or blackening of the different pizza samples as a function of the baking time (t_B_).

### 2.7. Statistical Analysis of Data

All data were listed in terms of average value ± standard deviation. The Tukey test was used to check for their statistically significant difference at a probability level (*p*) of 0.05. SYSTAT v. 8.0 (SPSS Inc., Chicago, IL, USA, 1998) was used to perform one-way analysis of variance.

## 3. Results and Discussion

Physically, pizza baking can be described as a process of simultaneous heat and liquid and vapor water transport within the product itself and within the gaseous environment inside the oven chamber. Conduction raises the temperature of the lower pizza surface, which is in contact with the hot oven floor, and then transfers heat from the lower surface to the upward layers of the crust, while radiation and convection transmit heat from the oven vault to the exposed upper surface of the pizza. Hence, these heat transfer mechanisms produce different localized heating effects, which will be monitored as reported below.

### 3.1. Assessment of the Different Area Sections of Baked Pizza Samples

By using the open-source image processing software ImageJ, it was possible to assess the surface area occupied by the ingredients used to top several pizza samples cooked in the pilot-scale wood-fired oven, as shown in Table 2.

Whatever the ingredient type and number used, there was no statistically significant difference among the overall surface areas of all the pizza samples tested at the 95% confidence level, this amounting to 623 ± 18 cm^2^, equivalent to an average diameter of 28.2 ± 0.4 cm. In addition, the surface area of the raised rim was independent of the garnishment used, with the average thickness of this annular section being equal to 2.2 ± 0.1 cm.

From Table 2, it can be noted that when using no ingredient (pizza A) or just one ingredient (tomato puree or sunflower oil), as in the case of pizza B and C, the internal surface area was practically constant (440 cm^2^), this representing about 71% of the overall pizza surface area. When using both these ingredients, the surface area covered by tomato puree or sunflower oil amounted to 48 or 23%, respectively. When the mozzarella cheese was further put in, the surface areas covered by sunflower oil, tomato puree, or mozzarella cheese totaled 7, 28, or 37% of the overall pizza surface area.

### 3.2. Monitoring of the Raised Rim Growth

**Figure 1:**
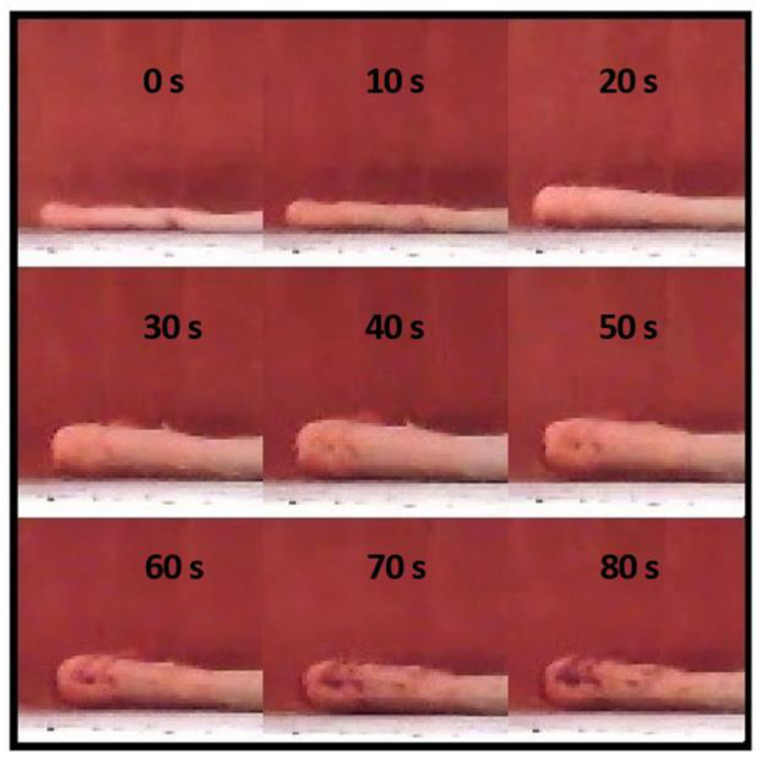
Cross section pictures of the pizza crust topped with tomato sauce and sunflower oil (pizza sample D: cf. Table 1) at different baking times in the range of 0 to 80 s.

During pizza baking, the heat received by the rim makes it expand because of local water evaporation. A thermal imaging camera was used to monitor the time course of its height (h) when baking different pizza samples of A–D type (Table 1), as shown for instance for the pizza sample D in Figure 1. An initial rapid growth of the edge occurred during the first 40 s, followed by a slower one in the following 40 s.

**Figure 2:**
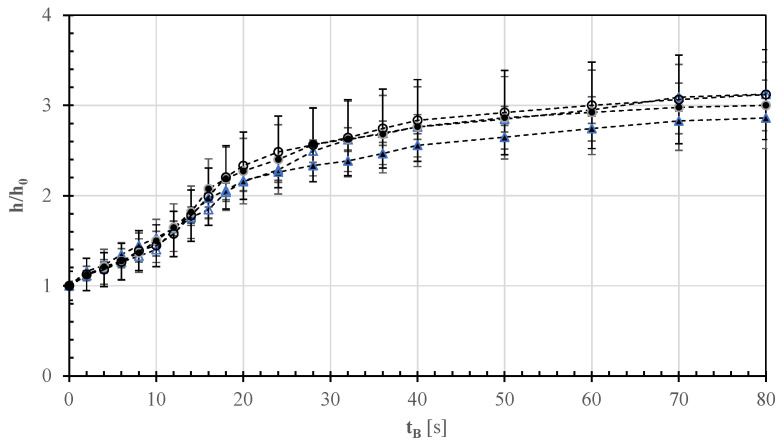
Effect of baking time (t_B_) on the average value and standard deviation of the ratio between the instantaneous (h) and initial (h_0_) heights of the raised rim of different pizza samples (A, ▲; B, △; C; ●; D, ○) during their baking in a pilot-scale wood-fired oven.

Table S1 in the electronic supplement shows the effect of baking time (t_B_) on the average value and standard deviation of the instantaneous height (h) of the raised rim of 15 different pizza samples of type A–D (cf. Table 1) during their baking in a pilot-scale wood-fired oven. The rim growth in white pizza samples (A) was not statistically different from that of tomato pizza samples (C) at a probability level of 0.05. This was also observed for the raised rims of white and tomato pizza samples both enriched with sunflower oil (B and D); however, these being statistically different from those of pizza samples of types A and C (Table S1). Taken together and accounting for an average data variability of 12%, the dimensionless ratio between the current (h) and initial (h0) heights of the raised rim appeared to be approximately independent of the garnishment ingredients used (Figure 2). Altogether, such a ratio increased from 1 to about 3 in as short as 80 s, while the rim height grew from 0.78 ± 0.09 cm to 2.33 ± 0.34 cm (Table S1). For this reason, its growth was assumed to be unrelated to the addition of a third ingredient (i.e., mozzarella cheese) in the internal pizza shell, and thus no further measurements were carried out for pizza E. As shown in Figure 2, the first exponential growth of the raised rim lasting about 20 s was followed by a linear growth during the subsequent 20–30 s, and then by declining growth during the remaining 30–40 s.

### 3.3. Mapping of the Thermal Profile of Pizza during Baking

Table S2 in the electronic supplement shows the mean values and standard deviations of the experimental temperatures of the oven floor exposed to fire and oven vault (T_FL_) or shielded by the pizza sample undergoing baking (T_FLbp_), and of different sectors of five pizza types (cf. Table 1), such as the raised rim (T_SR_), and upper (T_SU_) and lower (T_SL_) central areas, as baked in a wood-fired pizza oven operating in quasi steady-state conditions. Table S2 also shows the temperatures of the areas covered with tomato puree (TP) or sunflower oil (SO), with or without mozzarella cheese (MC), when 2 or 3 ingredients were distributed over the central area of the pizza shell. Each measurement was repeated 12 times for any of the five pizza types listed in Table 1.

**Figure 3:**
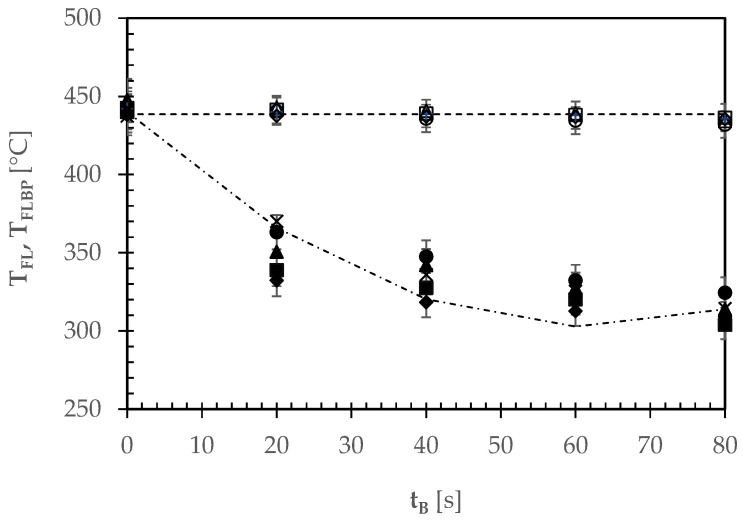
Time course of the average temperatures of the oven floor as exposed to fire (T_FL_: open and + symbols) or shielded by the pizza sample (T_FLbp_: closed and ✕ symbols) throughout the baking tests of different pizza types: A, ○, ●; B, △, ▲; C, □, ■; D, ◊, ◆; E, +, ✕. The horizontal broken line shows the average temperature of the oven floor around any pizza undergoing baking, while the dash-dotted line shows the quadratic regression line used to simulate the temperature profile of the oven floor under a tomato pizza (C).

Figure 3 shows the time course of the average temperatures of the oven floor as exposed to fire (T_FL_) or shielded by the pizza sample itself (T_FLbp_) throughout all the baking tests performed.

First, the oven floor temperature (T_FL_) exhibited no statistically significant variation around 439 ± 3 °C at the probability level *p* = 0.05, this confirming further that the oven was operating in quasi steady-state conditions. Second, the temperature of the oven floor at direct contact of each pizza showed a decreasing trend, that was accurately simulated by using a quadratic regression equation with coefficients of determination (r^2^) ranging from 0.98 to 0.99. The first derivate of T_FLbp_ with respect to t_B_ for t_B_ = 0 was expressed by a negative number, its modulus apparently increasing with the pizza mass. The greater the pizza mass per unit surface, the more rapid the cooling of the oven floor surface area over which the raw pizza was laid.

**Figure 4:**
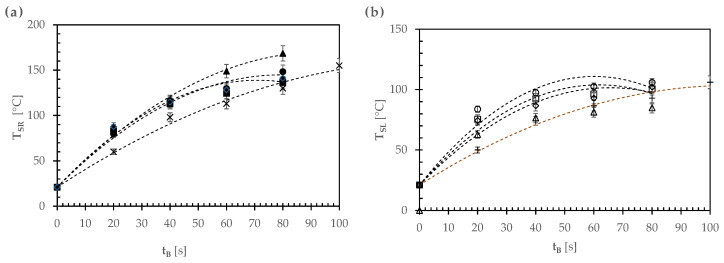
Time course of the average temperatures of (**a**) the raised rim (T_SR_: closed and ✕ symbols) and (**b**) lower area (T_SL_: open and + symbols) of all the pizza samples during the baking tests of different pizza types: A, ●, ○; B, ▲, △; C, ■, □; D, ◊, ◆; E, ✕, +. The broken lines were calculated using the specific least squares quadratic regressions.

Figure 4 shows the time course of the average temperatures of the raised rim (T_SR_) and lower area (T_SL_) of all the pizza samples fed into the wood-fired oven.

As shown in Figure 4a, after 80 s, the raised rim in all the pizza types under study increased to an average temperature (T_SR_) of 150 ± 13 °C, except for the margherita pizza (E) that reached such a temperature after 100 s owing to its greater mass (Table 1). All these thermal profiles were fitted using quadratic regression equations, their coefficients of determination (r^2^) ranging from 0.996 to 0.998 (see broken lines in Figure 4a). Moreover, in the case of pizza types A–D, for t_B_ = 0, (dT_SR_/dt_B_) and (d^2^T_SR_/dt_B_^2^) were approximately constant and equal to 3.2 ± 0.1 °C/s and −0.041 ± 0006 °C/s^2^, respectively. The final temperature of the raised rim was thus independent of the topping ingredients used and gave rise to quite a crispy area of the pizza crust.

The lower area of any pizza sample did not uniformly contact the hot oven floor owing to the presence of a laminar layer made of stagnant air and/or evaporated water. Thus, its temperature (T_SL_) increased up to an average value of 100 ± 9 °C in as short as 80 s, except for the pizza type E that reached such a temperature after 100 s (Figure 4b). By using the least squares method, quadratic regression equations were used to reconstruct the T_SL_ profiles, their coefficients of determination (r^2^) varying from 0.988 to 0.998 (see broken lines in Figure 4b). For the pizza types A–D, for t_B_ = 0, (dT_SL_/dt_B_) and (d^2^T_SL_/dt_B_^2^) were found to be approximately constant and equal to 2.7 ± 0.2 °C/s and −0.044 ± 0005 °C/s^2^, respectively. Probably, because of the pizzaiuolo’s ability at lifting and rotating the pizza toward the fire by means of a metal peel, not only was the pizza baked uniformly around its whole circumference, but the final temperature of the lower pizza area was also not so high as to burn it. This aspect will be further discussed below.

**Figure 5:**
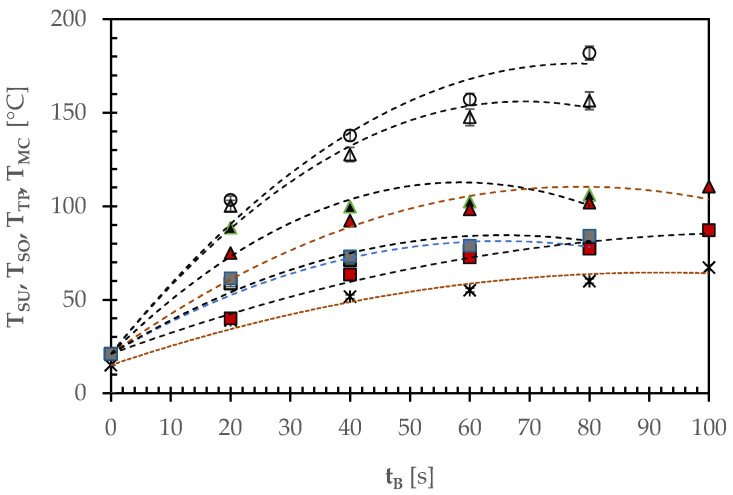
Time course of the average temperature of the upper area as a whole (T_SU_) or segmented with the two or three ingredients used to garnish the pizza samples examined in this work: A, ○; B, △; C, □; D: T_TP_,■; T_SO_, ▲; E: T_TP_, □; T_SO_, △; T_MC_, ✶), where T_TP_, T_SO_, or T_MC_ is the temperature of the pizza surface area garnished with tomato puree, sunflower oil, or mozzarella cheese, respectively. The broken lines refer to the quadratic regression lines used to simulate the different temperature profiles.

Figure 5 shows the time course of the average temperature (T_SU_) of the upper area of the pizza samples examined in this work. This temperature was related to the area devoid of any ingredient in the case of white pizza (A) or spread with sunflower oil (B) or tomato puree (C) only. In the case of pizza D, its central area having been spread with SO and TP, the thermal imaging camera was able to determine the average temperatures T_SO_ and T_TP_ of both areas. In the case of pizza E, the average temperatures of the areas covered with TP, SO, or mozzarella cheese pieces were measured.

At the end of baking, the temperature of the central upper side of white pizza (A) approached 182 ± 9 °C, probably because the formation of large dark brown colored areas increased the local emissivity and enhanced the absorption of the radiative heat from the oven vault. When the central upper area of white pizza was spread with sunflower oil (B), the increase in the pizza mass from 250 to 280 g limited its temperature increase to 156 ± 4 °C. For the pizzas D and E, the area covered with SO reached a lower temperature of 108 ± 3 °C, probably because of its smaller area exposed to the irradiating oven vault. When the whole central area of pizza C was garnished with tomato puree at 7 °Bx, its high moisture content limited the temperature growth to 81 ± 2 °C. Such a temperature was not statistically significantly different from that of the area equally topped with TP in pizza D or E, their average temperatures being equal to 84 ± 3 °C (Figure 5). Finally, the temperature of the area topped with white or pale ivory colored mozzarella cheese was definitively smaller (67 ± 2 °C), for its initial temperature (15 °C) was smaller than that (21 °C) of dough, TP, and SO, and emissivity lower than that of tomato puree.

### 3.4. Time Course of the Pizza Weight Loss

Table S2 lists the instantaneous mean mass (m_S_) of any pizza sample studied.

Such data were used to estimate the instantaneous amount of water evaporated during baking and thus calculate the current moisture mass fraction on an oil-free basis (x_W_) of the overall pizza sample (Table S2). It can be noted that the moisture content of white pizza such as (A) or topped with sunflower oil (B) reduced from 0.45 g/g to 0.43 or 0.42 g/g, respectively. On the contrary, x_W_ for the tomato pizza such as (C) or topped with SO (D) reduced from 0.555 to 0.542 g/g. The addition of MC in pizza sample E slightly affected x_W_, which lessened from 0.554 to 0.536 g/g.

**Figure 6:**
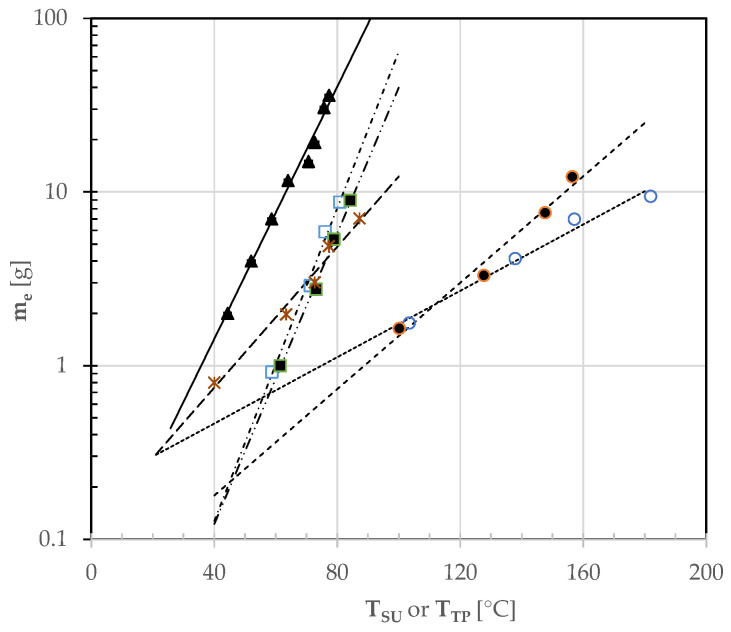
Semilogarithmic plot of the experimental amount of water evaporated (m_e_) against the average sample temperature (T_SU_ or T_TP_) measured during either the water heating test (▲, -) or different pizza baking tests (A: ○, …; B: ●, - - -; C: □, - . -; D: ■, —. .—; E: ✶, ^— —^. The different regressions lines were calculated using Equation (1) and the empirical coefficients listed in Table S3.

The amount of water evaporated (m_e_) during the baking tests carried out here was found to be a complex function of the average temperature of the sample, as well as its composition and water activity. When using no or just one topping ingredient, such a temperature was assumed as coincident with that of the upper side of the pizza crust (T_SU_). When the pizza was garnished with two or three ingredients, it was assumed as coincident with that of the surface area topped with tomato puree (T_TP_), this representing as much as 48 and 28% of the overall surface area of pizza types D and E, respectively. Thus, by plotting the m_e_ data collected during the water-heating [9] and pizza-baking tests against the sample temperature (T_S_) as specified above (i.e., T_SU_ or T_TP_) using a semi-logarithmic plot (Figure 6), it was possible to describe m_e_ via the following empirical relationship:

ln(m_e_) = a + b T_S_ (1)

where a and b are empirical coefficients that can be determined by using the least squares method, as shown in Table S3.

Obviously, water heating in aluminum trays having a diameter near to that of the pizza samples under study gave rise to greater water evaporation whatever the sample temperature. The samples C, D, and E, being all garnished with TP and having a greater moisture content around 0.55 g/g, exhibited a slower moisture evaporation. In pizza sample B, garnished with sunflower oil, water evaporation was even smaller. Nevertheless, at the end of their baking, such samples exhibited a higher temperature than that of samples C–E, this resulting in an overall weight loss greater than that of all the other pizza samples. Since the heat transferred by radiation and convection was almost constant [10], the low specific heat of sunflower oil allowed the pizza sample B to reach higher temperatures than that of the tomato puree area during baking, this enhancing the overall water vapor formation. Finally, the evaporation of sample A, being ungarnished, was exclusively related to the physical properties of the dough itself, which has a specific heat greater than sunflower oil but lower than tomato puree and mozzarella cheese. Altogether, at the end of baking, the overall amount of water evaporated was near to 10 g in spite of the different temperature of the upper side of the pizza types examined (Figure 6).

### 3.5. Color Visual Assessment of Baked Pizzas

The formation of brown or black colored areas in pizza during its baking in the wood-fired oven, due to the appearance of brown or black pigments, was previously monitored using computerized image analysis techniques and related to the available lysine content [13]. By using the IRIS electronic eye, any digital image was processed as a color spectrum on a maximum scale of 4096 colors, each of these corresponding to a unique set of three values in the RGB space. The black color was represented by the decimal code (0, 0, 0), while the brown one by (165, 42, 42), as derived from https://www.rapidtables.com/web/color/RGB_Color.html (accessed on 14 February 2023).

**Figure 7:**
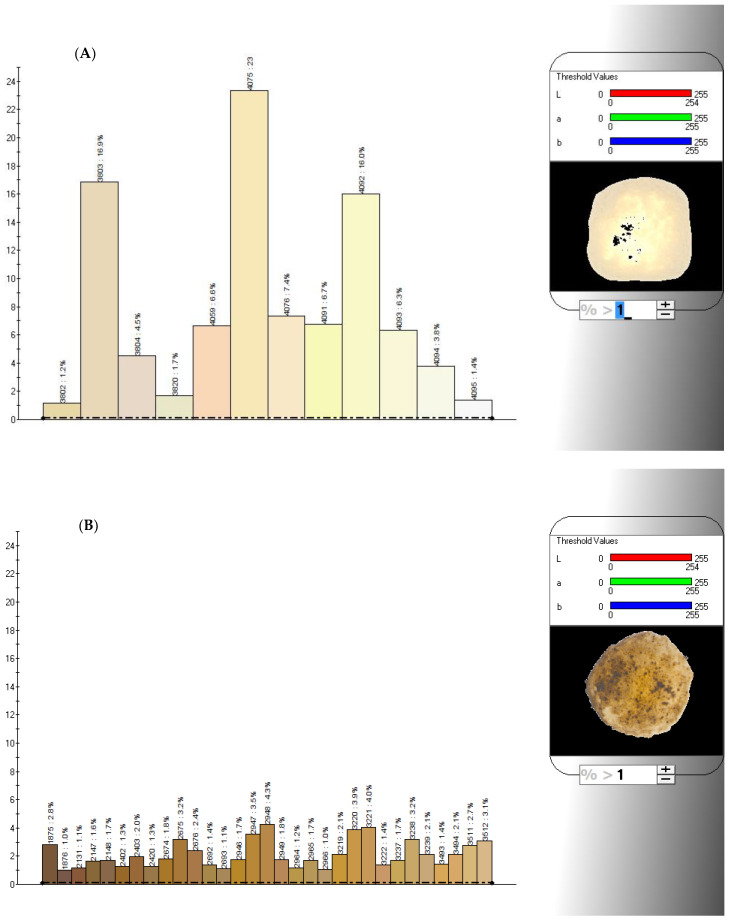
Color spectra of the upper side of pizza sample A (cf. Table 1) as freshly prepared (**A**) or after its baking in the pilot-scale wood-fired oven for 80 s (**B**), where the horizontal axis reports the color decimal code within a scale of 4096 colors and the vertical axis shows the percentage of the pizza surface area occupied by the corresponding color decimal code. The color spectra show the only colors occupying a percentage of the pizza surface area greater than 1%.

As an example, Figure 7 shows the color spectra of the pizza sample A as such and after 80 s baking in the pilot-scale wood-fired oven. By comparing such spectra, it was quite easy to highlight the color differences between these samples, as well as to quantify the area of each significant color and mark it as a percentage.

The effect of the browning or blackening process during the pizza baking was characterized by accounting for the color decimal codes seen as dark brown or black by the human eye. In particular, the browned areas of the pizza were characterized by 41 different decimal codes, while the blackened ones by 16 ones, as shown in Table 3. By associating such individual colors in two clusters, it was possible to derive the percentage of the pizza surface area denoted as browned (Br) or blackened (Bl).

Figure S2 in the electronic supplement shows the color spectra of the upper and lower sides of pizza samples A–E, as they were extracted from the oven after a baking time of 80 s for samples A–D or 100 s for the margherita pizza E; while Table S4 shows how the proportion of the browned or blackened area in both sides of such pizza samples increased as baking progressed.

As shown in Table S3, the percentage degree of browning or blackening in the lower pizza shell was smaller than that observed in the upper one. At the end of baking (t_B_ = 80 s), the central upper side of the white pizza sample (A) reached a temperature as high as 182 °C (Table S2), and thus exhibited the greatest Y_Br_ and Y_Bl_ values. Since the T_SU_ in pizza samples B was around 156 °C, its degree of browning was just near to 9%. In pizza samples C and D, the presence of tomato puree limited the temperature of the upper area to 81–84 °C, this resulting in a percentage of browning of about 11%, a value not statistically different from the above one at *p* = 0.05. Finally, pizza sample E was characterized by the smaller degree of browning (7.3%), probably because of the higher reflectivity of the mozzarella cheese pieces.

Concerning the degree of burning, its highest value was observed on the upper side of white pizza A (7.9%), even if the corresponding deviation standard, as high as 6%, made it not statistically different from those observed (1.4–3.9%) in the other pizza samples.

The degrees of browning and blackening on the lower side of all the pizza samples under study appeared to be unrelated not only to the use or not of topping ingredients, but also to the increase in the overall mass of each pizza. In principle, the greater the overall mass of the pizza, the more effective the contact between the pizza base and the hot oven floor will be. This should enhance the heat transfer through conduction from the bottom of the pizza and thus yield a more extensive blackening. This was in all probability counterbalanced by the pizzaiuolo’s ability at turning the pizza in almost the same area of the hot oven floor to limit or avoid burning the pizza bottom.

**Figure 8:**
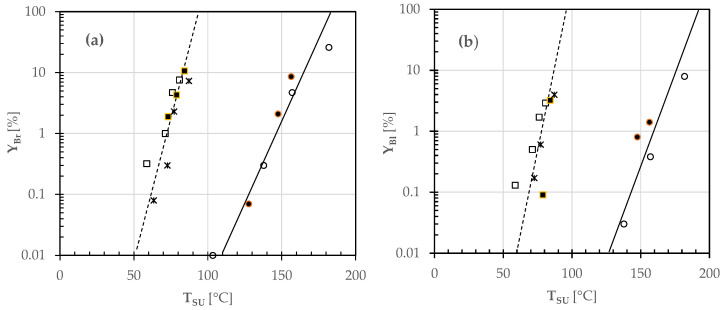
Semilogarithmic plot of the percentage degree of (**a**) browned (Y_Br_) and (**b**) blackened (Y_Bl_) areas of the upper surface area of different pizza samples (A: ○; B: ●; C: □; D: ■; E: ✶) during baking in a wood-fired oven versus the corresponding temperature (T_SU_). The continuous and broken lines were the least squares regression lines estimated using Equation (2).

**Figure 9:**
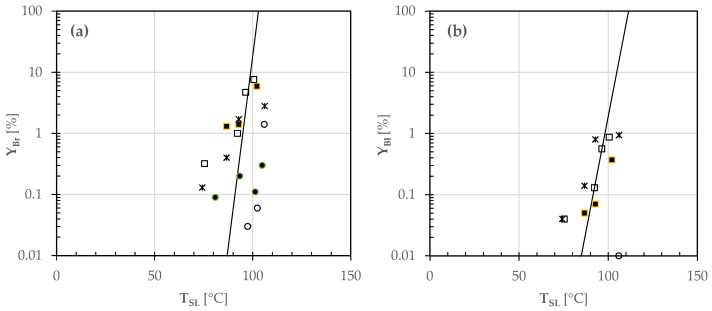
Semilogarithmic plot of the percentage degree of (**a**) browned (Y_Br_) and (**b**) blackened (Y_Bl_) areas of the lower surface area of different pizza samples (A: ○; B: ●; C: □; D: ■; E: ✶) during baking in a wood-fired oven versus the corresponding temperature (T_SL_). The continuous lines were the least squares regression lines estimated using Equation (2).

Although color formation in bakery products is caused by numerous parallel and consecutive reactions with various components, the appearance of brown pigments was generally simulated by assuming either zero order or first order kinetics [5,14,15]. To discriminate the mechanism of browning or blackening, the percentage degree Y_Br_ or Y_Bl_ versus the upper or lower pizza side temperature was plotted on a semilogarithmic scale, as shown in Figure 8 and Figure 9.

From Figure 8, it was observed that the curves of browning and burning on the upper surface area of all pizza samples might be described by straight lines on a semilogarithmic scale. Actually, two distinct straight lines were identified, the first one fitting the color change of white pizzas such as (A) or topped with sunflower oil (B), and the second one that of tomato pizzas such as (C) or garnished with SO only (D) or with mozzarella cheese also (E). From Figure 9, the browning and burning yields for all the pizza samples under study were scattered, so were roughly fitted using a single straight line. 

In the circumstances, the experimental Y_Br_ and Y_Bl_ data were reconstructed according to Bigelow et al. [16]:

(2) $$\log\frac{Y_{i}}{Y_{\mathrm{iR}}}=\frac{T_{\mathrm{Sj}}-T_{\mathrm{SjR}}}{z_{i}}$$

where Y_i_ is the percentage degree of browning (Br) or blackening (Bl) corresponding to the actual (T_Sj_) and reference (T_SjR_) temperatures of the upper or lower side of any pizza sample, and z_i_ is the temperature increment needed for a ten-fold acceleration of the rate of pizza browning or blackening (i.e., for increasing Y_i_ by a factor of 10).

By using the least squares method, it was possible to fit the experimental Y_i_ values, as shown by the continuous and broken lines plotted in Figure 8 and Figure 9. Table 4 lists the empirical coefficients (z_i_ and T_SjR_) of the least-squares regressions.

In the literature, such a first-order kinetic model has been generally used to describe the death rate of free cells and spores, as well as the inactivation or degradation rate of enzymes, vitamins, and pigments [17]. Whereas the z values characterizing microbial death ranged from 5 to 11 °C, those related to enzyme inactivation varied from 15 to 20 °C [18], and those concerning typical chemical reactions, such as vitamin B1 and chlorophyll destruction [17], or the optimal cooking time of different pasta formats [19], were found to fluctuate from 25 to 111 °C.

In this case, the formation rate of browned or blackened areas in baked pizza was increased 10-fold as the temperature of the upper side of pizza was increased by 19 or 16 °C in the case of white pizzas A and B, or by about 9 °C in the case of any tomato pizza (C–E). This might be the result of the inertial effect exerted by the addition of an aqueous-rich tomato puree. In fact, the moisture content of white pizzas was definitely smaller than that of tomato pizzas (Table S2). On the contrary, there was no statistically significant difference between the z values characterizing the temperature-sensitivity of the lower side of any white or tomato pizzas to browning and burning, probably because of the highly scattered data collected.

In the circumstances, whatever the pizza type baked, the percentage of burning of its bottom was generally far smaller than that observed on its upper side. This definitively contradicts the general belief that the bottom of pizza baked in wood-fired ovens is more burnt than that cooked in gas or electric ovens. Since the blackened areas observed in tomato pizzas covered up to 4% of total pizza surface areas (Table S4), their wastage would be lower than the amount (~6%) of pizza averagely discarded at the end of a meal in a typical Neapolitan pizzeria [20]. This would avoid the health risk of ingesting charred pizza pieces with high levels of acrylamide, its accumulation in starchy foods baked, fried, or roasted at 120–150 °C increasing the risk of developing cancer for consumers in all age groups [21]. In fact, despite the Food Safety Authority (EFSA) recommendation to not exceed the dose of 0.17 mg of acrylamide per kg of body weight and day [22], the concentration of acrylamide in pizzas baked in a wood-fired oven was found to range from 0.8 to 2.4 mg/kg [23].

## 4. Conclusions

In this work, Neapolitan pizza baking in a pilot-scale wood-fired oven operating in quasi steady-state conditions was phenomenologically analyzed by using color visual analysis and IR thermal scanning.

First, at the end of baking, all pizza samples tested had almost the same diameter (28.2 ± 0.4 cm) and a raised rim, 2.2 cm in thickness and 2.3 cm in height, whatever the topping ingredients used.

During pizza baking, the oven floor temperature did not change, being practically constant at 439 ± 3 °C; while the area underneath each pizza reduced its temperature faster the greater the pizza mass laid on it. The pizza bottom reached a maximum temperature of 100 ± 9 °C, the pizzaiuolo being quite skilled at lifting and rotating the pizza to bake it uniformly around its whole circumference. By contrast, the upper pizza side was heated up to 182, 84, or 67 °C in the case of white pizza, tomato pizza, or margherita pizza, respectively, mainly because of their diverse moisture content and emissivity. The water vapor weight loss was nonlinearly related to the average temperature of the upper pizza shell when using no or just one topping ingredient, or that of tomato puree-topped surface area. In all pizza types examined, the overall water vapor weight loss was near to 10 g. The formation of brown or black colored areas in the upper and lower sides of baked pizza was detected with the help of the IRIS electronic eye using 41 or 16 different decimal color codes in the RGB color space, these being denoted as dark brown or black, respectively. The upper pizza side exhibited greater degrees of browning and blackening than the lower one, with maximum values of about 26 and 8% being observed, respectively, in white pizza. The formation rate of browned or blackened areas was described via the Bigelow first-order kinetic model and was characterized by a ten-fold increase as the temperature of the upper side of the pizza was raised by 16–19 °C or by about 9 °C in the case of any white or tomato pizzas, respectively. However, such a kinetic model was unable to describe the temperature-sensitivity of all pizza bottoms.

Altogether, the above results should have both economic and scientific relevance. The right cooking of pizza involves the formation of a well-developed rim with quite limited blackened surface areas. Moreover, the moisture content of the crust has to be appropriately lowered so that the pizza retains its flexibility, but it is no way toasted. These aspects are fundamental in qualitative and economic terms, as highlighted and discussed previously [3]. From the only scientific point of view, it is worth pointing out that the very rapid pizza baking in a wood-fired oven, or alternatively in an electric or gas oven, is one of the steps characterizing the art of the Neapolitan pizza maker. Such a step implies the right management of the heat and mass transfer processes that take place during baking, whose kinetics affect the sensory characteristics of the Neapolitan pizza. Although the Neapolitan pizza is a product widespread all over the world, no other analysis of pizza baking phenomenology is currently available in the scientific literature. In this way, the main results of this work could help develop a specific modelling and monitoring strategy to reduce variability and maximize the quality attributes of Neapolitan pizza.

## Figures and Tables

**Table 1:** Samples of Neapolitan pizza submitted to baking tests in the wood-fired oven used here.

Sample	Topping	Overall Mass [g]
A	No garnishment	250 ± 1
B	Sunflower oil (30 g)	280 ± 2
C	Tomato puree (70 g)	320 ± 2
D	Tomato puree (70 g) and sunflower oil (30 g)	350 ± 3
E	Tomato puree (70 g), sunflower oil (30 g), and Mozzarella cheese (80 g)	430 ± 5

**Table 2:** Overall and partial areas of the pizza base as garnished with 1, 2, or 3 ingredients (SO, sunflower oil; TP, tomato puree; MC, mozzarella cheese), together with its average diameter and thickness of the raised rim.

Topping Ingredient	No.	0	1	1	2	3
	Type	-	SO	TP	SO + TP	SO + TP + MC
Pizza Type		A	B	C	D	E
	Unit	Mean ± sd	Mean ± sd	Mean ± sd	Mean ± sd	Mean ± sd
Rim Area	cm^2^	182 ± 9 ^a^	182 ± 12 ^a^	179 ± 5 ^a^	181 ± 9 ^a^	180 ± 11 ^a^
SO Area	cm^2^	-	441 ± 25 ^a^	-	141 ± 24 ^b^	43 ± 5 ^c^
TP Area	cm^2^	-	-	440 ± 17 ^a^	302 ± 8 ^b^	172 ± 21 ^c^
MC Area	cm^2^	-	-	-	-	232 ± 13
Overall Area	cm^2^	620 ± 18 ^a^	623 ± 14 ^a^	619 ± 12 ^a^	624 ± 24 ^a^	624 ± 24 ^a^
Pizza Diameter	cm	28.1 ± 0.4 ^a^	28.2 ± 0.3 ^a^	28.1 ± 0.3 ^a^	28.2 ± 0.5 ^a^	28.3 ± 0.7 ^a^
Average Rim Thickness	cm	2.2 ± 0.1 ^a^	2.2 ± 0.2 ^a^	2.2 ± 0.1 ^a^	2.2 ± 0.2 ^a^	2.2 ± 0.2 ^a^

In each row, values with the same letter have no significant difference at *p* < 0.05.

**Table 3:** Decimal color codes associated with the browned and blackened areas of a pizza undergoing baking in a wood-fired oven.

Pizza Area	Color Decimal Code												
Browned	1857	1858	1859	1873	1874	1875	1876	1891	1892	1893	1894	2128	2129
	2130	2131	2132	2145	2146	2147	2148	2149	2165	2166	2400	2401	2402
	2403	2404	2405	2417	2418	2419	2420	2421	2422	2438	2657	2658	2659
	2672	2673											
Blackened	1075	1091	1092	1331	1346	1347	1348	1364	1365	1602	1603	1604	1618
	1619	1620	1621										

**Table 4:** Least squares estimate of the empirical coefficients (z_i_, T_SiR_ and Y_iR_) of Equation (2), as referred to the browned and blackened degrees of different pizza samples undergoing baking in a wood-fired oven, and corresponding coefficients of determinations (r^2^).

Browning or Burning Kinetics	T_SiR_ [°C]	z_i_ [°C]	Y_iR_ [%]	r^2^
Browning of the upper pizza side				
White pizza A and B	100	19 ± 3	0.0032	0.90
Tomato pizza C, D, and E	50	8 ± 3	0.0021	0.41
Burning of the upper pizza side				
White pizza A and B	100	16 ± 5	0.00024	0.79
Tomato pizza C, D, and E	50	9 ± 4	0.0009	0.48
Browning of the lower pizza side				
Pizza A–E	100	4 ± 3	18.3	0.08
Burning of the lower pizza side				
Pizza A–E	100	5 ± 5	1.92	0.17

## Data Availability

All data are reported in this article.

## Supplementary Materials

The following supporting information can be downloaded at: https://www.mdpi.com/article/10.3390/foods12040890/s1, Figure S1: Front picture of the wood-fired pizza oven; Figure S2: Color spectra of the upper and lower sides of baked pizza samples A–E; Table S1: Time course of the instantaneous raised rim height of different pizza samples undergoing baking; Table S2: Time course of the instantaneous temperatures of the oven floor and different areas of the pizza samples A–E undergoing baking; Table S3: Empirical coefficients a and b of Equation (1); Table S4: Time course of the percentage degree of browned and blackened areas of the upper and lower area of different pizza samples A–E during baking in a wood-fired oven.
