# Supplementary material for: Phenomenology of Neapolitan Pizza Baking in a Traditional Wood-Fired Oven
Source: Foods. 2023 Feb 19;12(4):890. doi: 10.3390/foods12040890 (PMC9957212; doi:10.3390/foods12040890)

Phenomenology of Neapolitan pizza baking in a traditional wood-fired oven

Aniello FALCIANO [1,2], Mauro MORESI [3*] and Paolo MASI [1,2]

[1] Department of Agricultural Sciences, University of Naples Federico II, Portici, Italy
[2] Academic Centre for Food Innovation, University of Naples Federico II, Portici, Italy
[3] Department for Innovation in the Biological Agrofood and Forestry Systems, University of Tuscia, Viterbo, Italy

Table S1 – Effect of baking time (t_B) on the average value and standard deviation of the instantaneous height (h) of the rim of different pizza samples (see types A-D in Table 1) during their baking in a pilot-scale wood-fired oven.

Rim height (h) of pizza sample [cm] t_B [s]	A	B	C	D
0	0.68±0.11^a	0.85±0.14^b	0.74±0.20^a	0.87±0.17^b
2	0.78±0.14^a	0.94±0.16^b	0.82±0.18^a	0.96±0.16^b
4	0.84±0.18^a	1.01±0.19^b	0.88±0.17^a	1.01±0.16^b
6	0.91±0.18^a	1.07±0.20^b	0.93±0.19^a	1.08±0.20^b
8	0.98±0.21^a	1.12±0.21^b	0.99±0.20^a	1.19±0.23^b
10	1.04±0.21^a	1.18±0.22^{a,b}	1.09±0.22^a	1.23±0.24^b
12	1.11±0.22^a	1.37±0.22^b	1.20±0.25^a	1.34±0.23^b
14	1.21±0.26^a	1.47±0.22^b	1.31±0.22^a	1.51±0.28^b
16	1.34±0.23^a	1.55±0.25^b	1.48±0.21^a	1.68±0.35^b
18	1.40±0.28^a	1.72±0.33^b	1.57±0.22^a	1.87±0.42^b
20	1.47±0.33^a	1.82±0.37^b	1.62±0.21^a	1.98±0.43^b
24	1.54±0.34^a	1.92±0.41^b	1.71±0.24^a	2.11±0.47^b
28	1.59±0.37^a	2.10±0.47^b	1.83±0.28^a	2.17±0.47^b
32	1.63±0.39^a	2.21±0.45^b	1.88±0.29^a	2.24±0.47^b
36	1.69±0.42^a	2.26±0.42^b	1.92±0.31^a	2.32±0.49^b
40	1.75±0.45^a	2.32±0.42^b	1.97±0.33^a	2.40±0.50^b
50	1.81±0.49^a	2.39±0.38^b	2.04±0.35^a	2.47±0.50^b
60	1.88±0.52^a	2.47±0.36^b	2.08±0.36^a	2.53±0.50^b
70	1.93±0.50^a	2.58±0.34^b	2.12±0.35^a	2.58±0.45^b
80	1.96±0.50^a	2.61±0.33^b	2.14±0.35^a	2.63±0.45^b

In each row, values with the same letter have no significant difference at $p < 0.05$.

Table S2: Main results (mean ± sd) of 12 repeated baking tests performed in a wood-fired pizza oven fed with 3 kg/h of oak logs using five pizza types A- E (see Table 1): effect of baking time (t_B) on the instantaneous temperature of the oven floor exposed to fire (T_{FL}) or shielded by the pizza sample (T_{FLbp}), temperatures of the pizza rim (T_{SR}), upper (T_{SU}) and lower (T_{SL}) areas, overall mass of sample (m_S), and estimated moisture fraction on oil-free basis (x_W). When 2 o 3 ingredients were added, T_{SU} was expressed by averaging the temperatures of the areas covered with tomato puree (TP), sunflower oil (SO) and/or mozzarella cheese (MC).

t_B	T_{FL}	T_{FLbp}	T_{SR}	T_{SU}			T_{SL}	m_S	x_W
[s]	[°C]	[°C]	[°C]	[°C]			[°C]	[g]	[g/g]
White pizza									
0	442 ± 9 ^{a}	442 ± 9 ^{a}	21.0±0.1 ^{a}	21.0±0.1 ^{a}			21.0±0.1 ^{a}	250.0±1.0 ^{a}	0.450
20	441 ± 7 ^{a}	363 ±10 ^{b}	80.0±3.0 ^{b}	103.0±2.0 ^{b}			84.0±2.0 ^{b}	248.2±0.2 ^{b}	0.446
40	436 ±11 ^{a}	348 ± 5 ^{b}	116.0±3.0 ^{c}	138.0±7.0 ^{c}			97.0±2.0 ^{c}	245.9±0.6 ^{c}	0.440
60	435 ± 7 ^{a}	332 ± 7 ^{c}	130.0±6.0 ^{d}	157.0±6.0 ^{d}			102.0±2.0 ^{d}	243.0±1.0 ^{d}	0.434
80	432 ±10 ^{a}	325 ± 5 ^{c}	148.0±9.0 ^{e}	182.0±9.0 ^{e}			106.0±3.0 ^{d}	240.6±0.7 ^{e}	0.428
White pizza garnished with sunflower oil									
0	446 ± 5 ^{a}	448 ± 7 ^{a}	21.0±0.1 ^{a}	21.0±0.1 ^{a}			21.0±0.1 ^{a}	280.0±2.0 ^{a}	0.450
20	443 ± 6 ^{a}	351 ±11 ^{b}	86.0±3.0 ^{b}	100.0±3.0 ^{b}			81.0±2.0 ^{b}	278.4±0.2 ^{a}	0.446
40	441 ± 7 ^{a}	342 ± 9 ^{b}	116.0±7.0 ^{c}	128.0±6.0 ^{c}			93.0±5.0 ^{c}	276.7±0.6 ^{b}	0.442
60	439 ±11 ^{a}	327 ± 7 ^{c}	149.0±7.0 ^{d}	148.0±5.0 ^{d}			101.0±3.0 ^{d}	272.4±1.3 ^{c}	0.432
80	434 ± 8 ^{a}	314 ± 7 ^{b,c}	169.0±9.0 ^{e}	156.0±4.0 ^{d}			105.0±2.0 ^{d}	267.7±1.6 ^{d}	0.421
Tomato pizza									
0	443 ± 8 ^{a}	440 ± 7 ^{a}	21.0±0.1 ^{a}	21.0±0.1 ^{a}			21.0±0.1 ^{a}	320.0±2.0 ^{a}	0.555
20	442 ± 7 ^{a}	339 ±10 ^{b}	83.0±2.0 ^{b}	59.0±2.0 ^{b}			75.0±2.0 ^{b}	319.1±0.3 ^{a}	0.553
40	439 ± 7 ^{a}	328 ± 6 ^{b}	113.0±4.0 ^{c}	71.0±2.0 ^{c}			92.0±3.0 ^{c}	317.1±0.5 ^{b}	0.551
60	438 ± 8 ^{a}	320 ±10 ^{b,c}	124.0±3.0 ^{d}	76.0±2.0 ^{d}			96.0±2.0 ^{c}	314.1±0.3 ^{c}	0.546
80	436 ± 6 ^{a}	304 ± 5 ^{c}	136.0±3.0 ^{e}	81.0±2.0 ^{e}			101.0±2.0 ^{d}	311.2±0.8 ^{d}	0.542
Tomato pizza garnished with sunflower oil									
				TP area	SO area				
0	440 ± 7 ^{a}	438 ±10 ^{a}	21.0±0.1 ^{a}	21.0±0.1 ^{a}	21.0±0.1^{a}		21.0±0.1 ^{a}	350.0±3.0 ^{a}	0.555
20	438 ± 5 ^{a}	332 ±12 ^{b}	88.0±3.0 ^{b}	61.0±3.0 ^{b}	89.0±5.0^{b}		74.0±3.0 ^{b}	349.4±0.1 ^{a}	0.554
40	437 ± 7 ^{a}	318 ± 5 ^{b,c}	115.0±5.0 ^{c}	73.0±2.0 ^{c}	100.0±4.0^{c}		87.0±2.0 ^{c}	347.2±0.5 ^{b}	0.551
60	437 ± 6 ^{a}	313 ± 7 ^{b,c}	128.0±5.0 ^{d}	79.0±2.0 ^{d}	103.0±2.0^{c}		93.0±2.0 ^{d}	344.7±0.3 ^{c}	0.547
80	436 ± 6 ^{a}	309 ± 7 ^{c}	141.0±2.0 ^{e}	84.0±2.0 ^{e}	106.0±2.0^{c}		102.0±2.0 ^{e}	341.0±1.9 ^{d}	0.542
Tomato pizza garnished with sunflower oil and mozzarella cheese									
				TP area	SO area	MC area			
0	442 ± 9 ^{a}	437 ±12 ^{a}	21 ± 0.1 ^{a}	21.0±0.1 ^{a}	21.0±0.1^{a}	15.0±0.1^{a}	21.0±0.1 ^{a}	430.0±4.0 ^{a}	0.554
40	439 ± 4 ^{a}	336 ±10 ^{b}	98 ± 3 ^{b}	63.0±2.0 ^{b}	92.0±4.0 ^{b}	51.6±1.8 ^{b}	74.3±2.6 ^{b}	428.0±0.6 ^{a}	0.542

60	438 ± 7 ^{a}	325 ± 6 ^{b,c}	113 ± 3 ^{c}	73.0±2.0 ^{c}	98.0±3.0 ^{c}	55.0±2.0 ^{c}	86.7±2.0 ^{c}	427.0±0.6 ^{b}	0.540
80	436 ± 6 ^{a}	314 ± 7 ^{b,c}	130 ± 5 ^{d}	77.0±3.0 ^{c}	101.0±2.0 ^{c}	59.9±1.6 ^{d}	92.8±2.1 ^{d}	425.1±0.6 ^{c}	0.538
100	436 ± 5 ^{a}	307 ± 6 ^{c}	155 ± 5 ^{e}	87.0±2.0 ^{c}	110.6±3.4^{d}	67.2±2.4 ^{e}	106.1±3.7 ^{e}	423.0±0.3 ^{d}	0.536

Mean values within the same parameter at different baking times followed by different superscript letters significantly differ by the Tukey test ($p<0.05$).

Table S3 Mean value and standard deviation of the empirical coefficients **a** and **b** of Eq. (1) and corresponding coefficient of determinations (r^2) for the water heating and pizza baking tests carried out in this work.

**Sample**	**a**	**b**	$\mathbf{r^2}$
Water	-2.99±0.26	0.084±0.004	0.987
A) Pizza as such	-1.65±0.34	0.022±0.002	0.979
B) Pizza topped with SO	-3.13±0.52	0.035±0.004	0.977
C) Pizza topped with TP	-6.22±0.48	0.104±0.007	0.992
D) Pizza topped with TS and SO	-5.96±0.31	0.097±0.004	0.996
E) Pizza topped with TS, SO, and MC	-2.16±0.27	0.047±0.004	0.980

Table S4

Effect of baking time (t_B) on the percentage degree of browned (Y_{Br}) and blackened (Y_{Bl}) areas of the upper and lower area of different pizza samples A-E (cf. Table 1) during baking in a wood-fired oven. Each percentage is expressed as mean ± sd (n = 3).

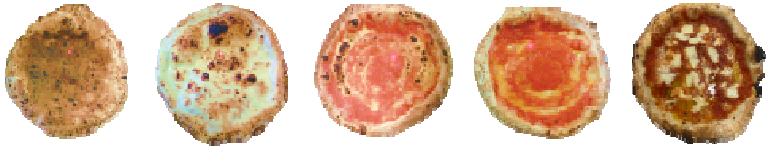

**Pizza sample**	**A**	**B**	**C**	**D**	**E**	**A**	**B**	**C**	**D**	**E**
**t_B (s)**	**Browned area percentage Y_{Br} [%]**					**Blackened area percentage Y_{Bl} [%] (%)**				
Upper pizza side										
20	0.01±0.0	0.00±0.0	2.5±1.0			0.00±0.0	0.00±0.0	0.13±0.2		
40	0.3±0.2	0.07±0.1	3.1±1.2	1.9±0.3	0.08±0.1	0.03±0.1	0.00±0.0	0.5±0.3	0.00±0.0	0.00±0.0
60	4.7±1.0	2.1±1.5	8.2±2.0	4.3±0.5	0.3±0.1	0.38±0.1	0.8±1.7	1.7±0.8	0.09±0.1	0.17±0.0
80	26±5 ^{a}	8.6±1.6 ^{c,b}	11±2 ^{b}	10.7±5 ^{b}	2.3±0.1	7.9±6 ^{a}	1.4±1.1 ^{a}	2.9±0.1 ^{a}	3.2±2.0 ^{a}	0.6±0.1
100					7.3±0.3 ^{c}					3.95±0.3 ^{a}
Lower pizza side										
20	0.00±0.0	0.09±0.1	0.32±0.3			0.00±0.0	0.00±0.0	0.04±0.0		
40	0.03±0.0	0.2±0.3	1.0±0.4	1.3±0.9	0.13±0.0	0.00±0.0	0.00±0.0	0.13±0.1	0.05±0.0	0.04±0.0
60	0.06±0.1	0.11±0.5	4.7±1.7	1.4±1.4	0.40±0.0	0.00±0.0	0.00±0.0	0.56±0.1	0.07±0.0	0.14±0.0
80	1.4±1.2 ^{b}	0.3±0.2 ^{a}	7.6±1.2 ^{c}	5.9±1.0 ^{c}	1.7±0.1	0.01±0.0 ^{c}	0.00±0.0 ^{c}	0.87±0.4 ^{a,b}	0.37±0.2 ^{b}	0.80±0.1
100					2.8±0.1 ^{b}					0.94±0.1 ^{a}

Mean values within the same parameter at different baking times followed by different superscript letters significantly differ by the Tukey test ($p<0.05$).

Figure S1
Front picture of the wood-fired pizza oven used in this work.

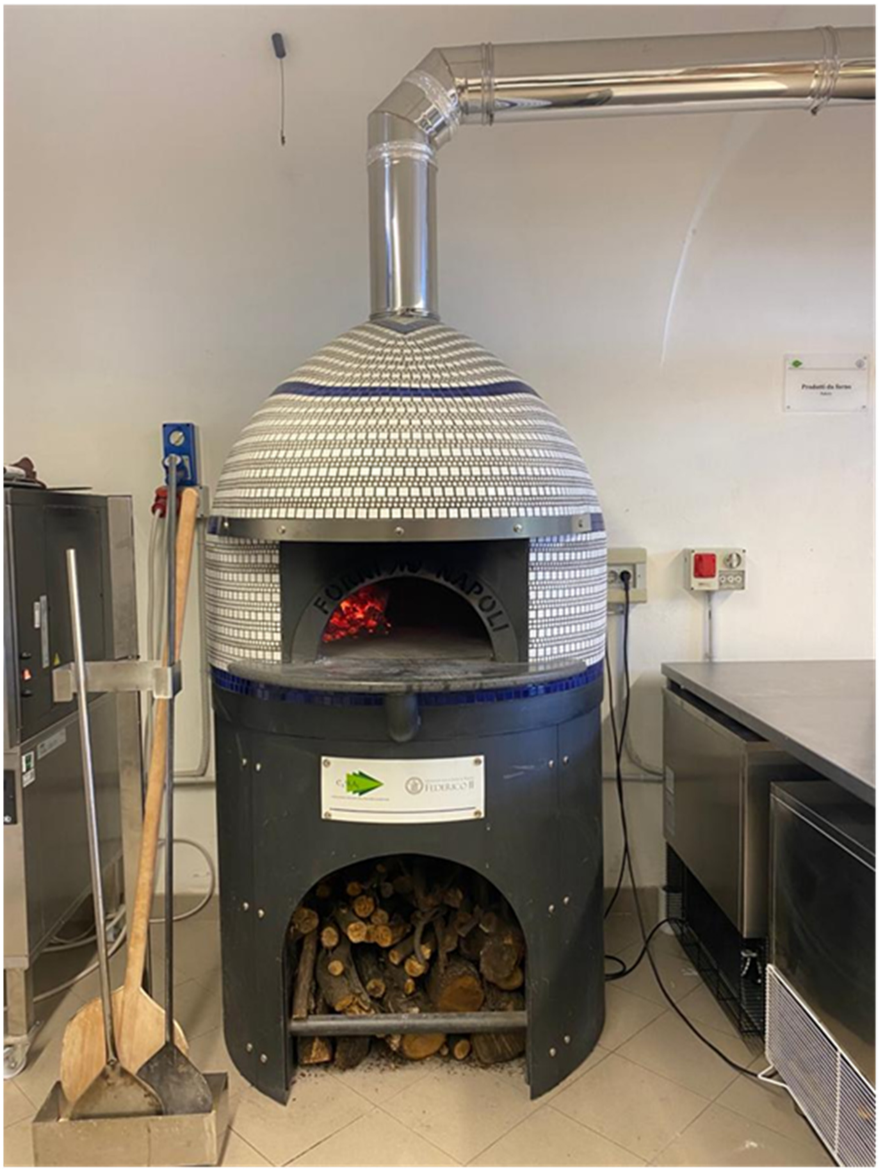

Figure S2

Color spectra of the upper and lower sides of pizza samples A-E (cf. Table 1) as baked in the pilot-scale wood-fired oven for 80 s showing the proportion (percentage of surface) of each unique color measured in a 4096-color space if greater than 0.1%.

a) Upper side of pizza sample A

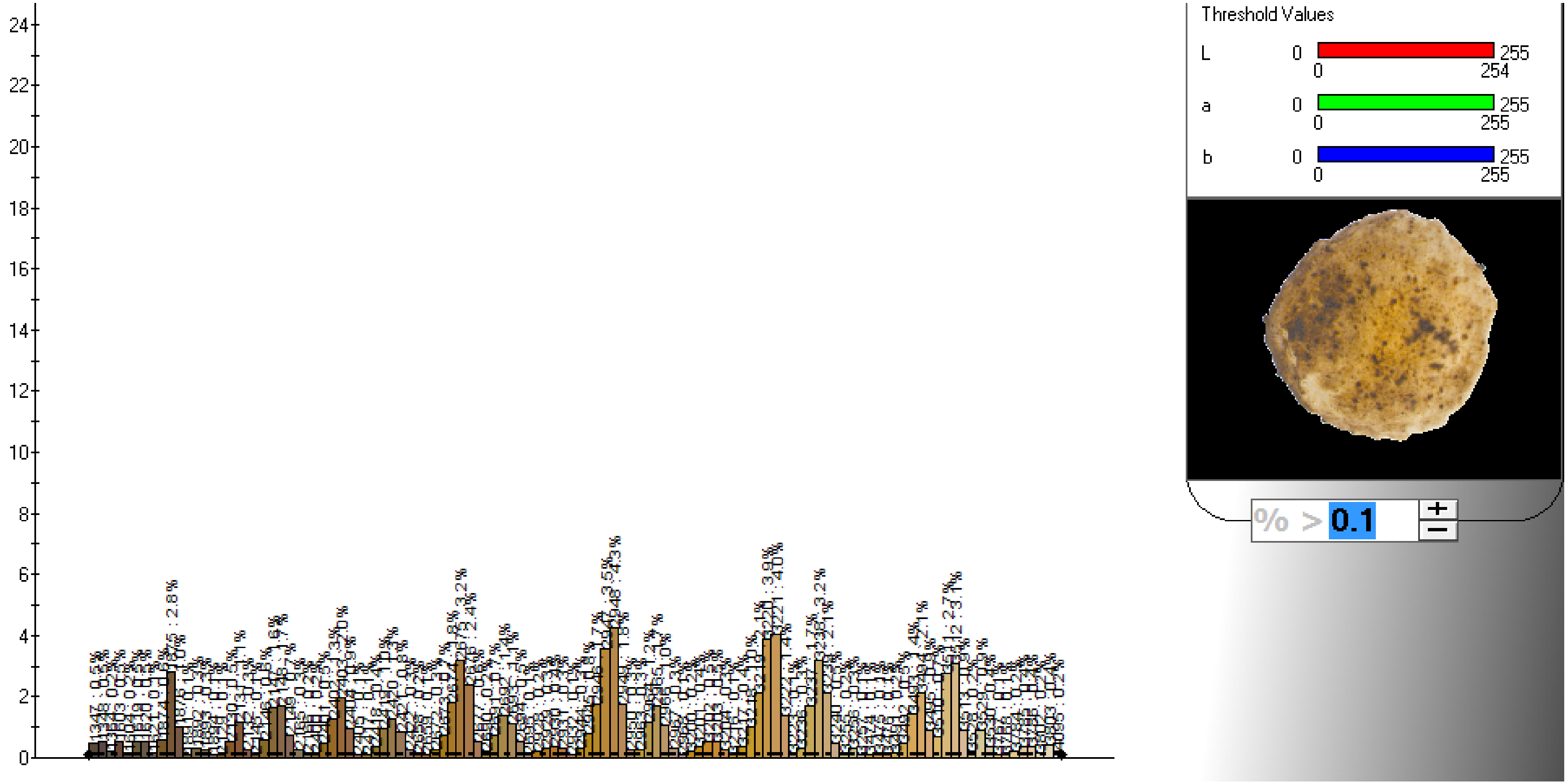

b) Lower side of pizza sample A

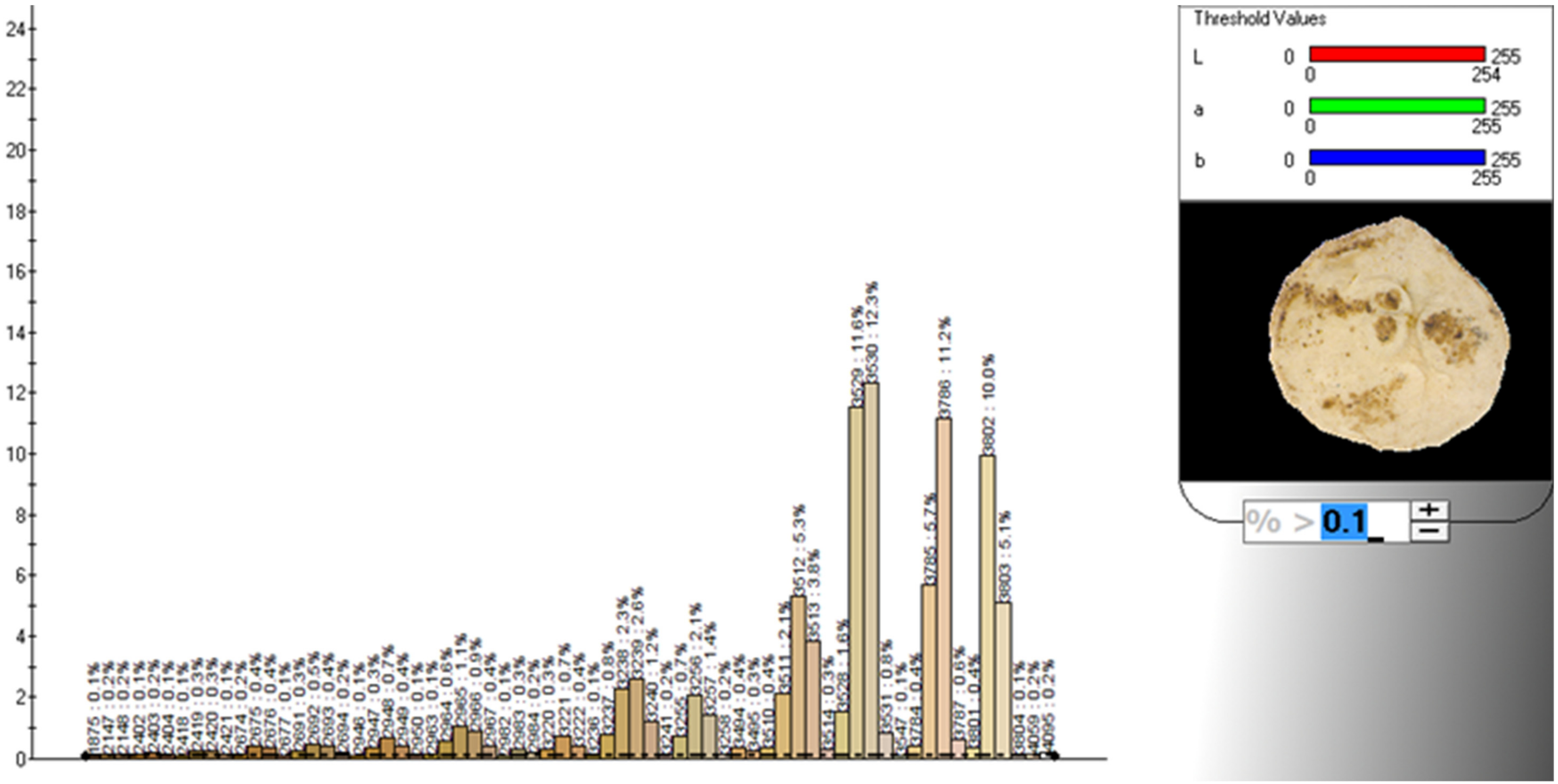

c) Upper side of pizza sample B

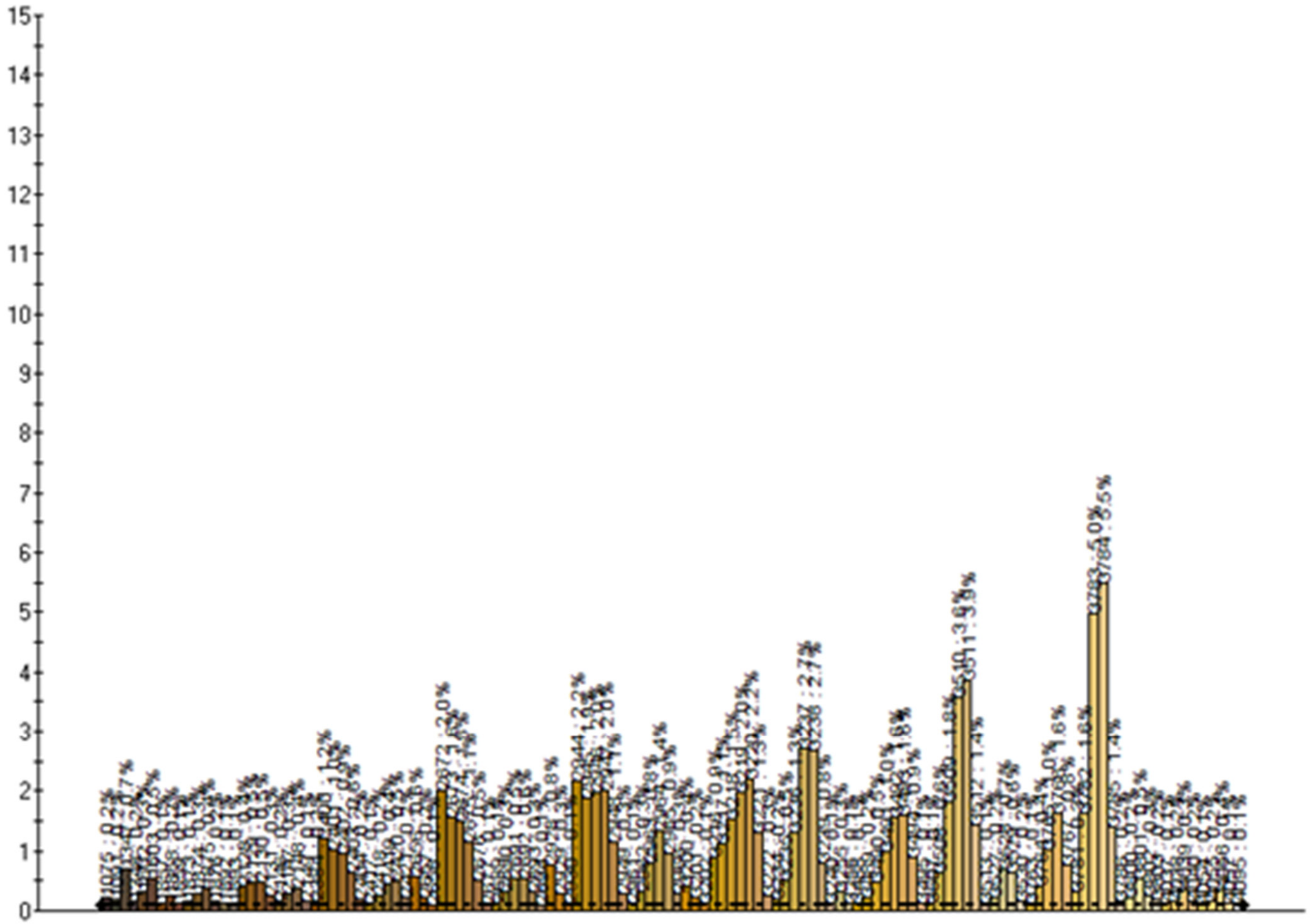

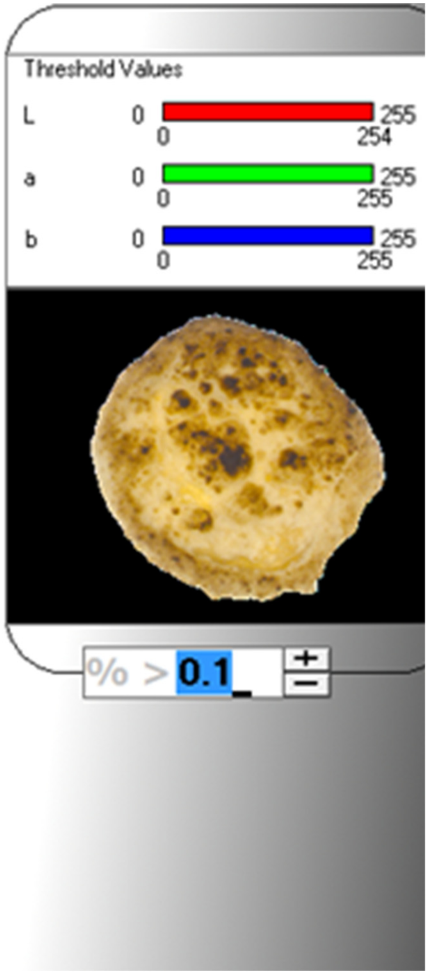

d) Lower side of pizza sample B

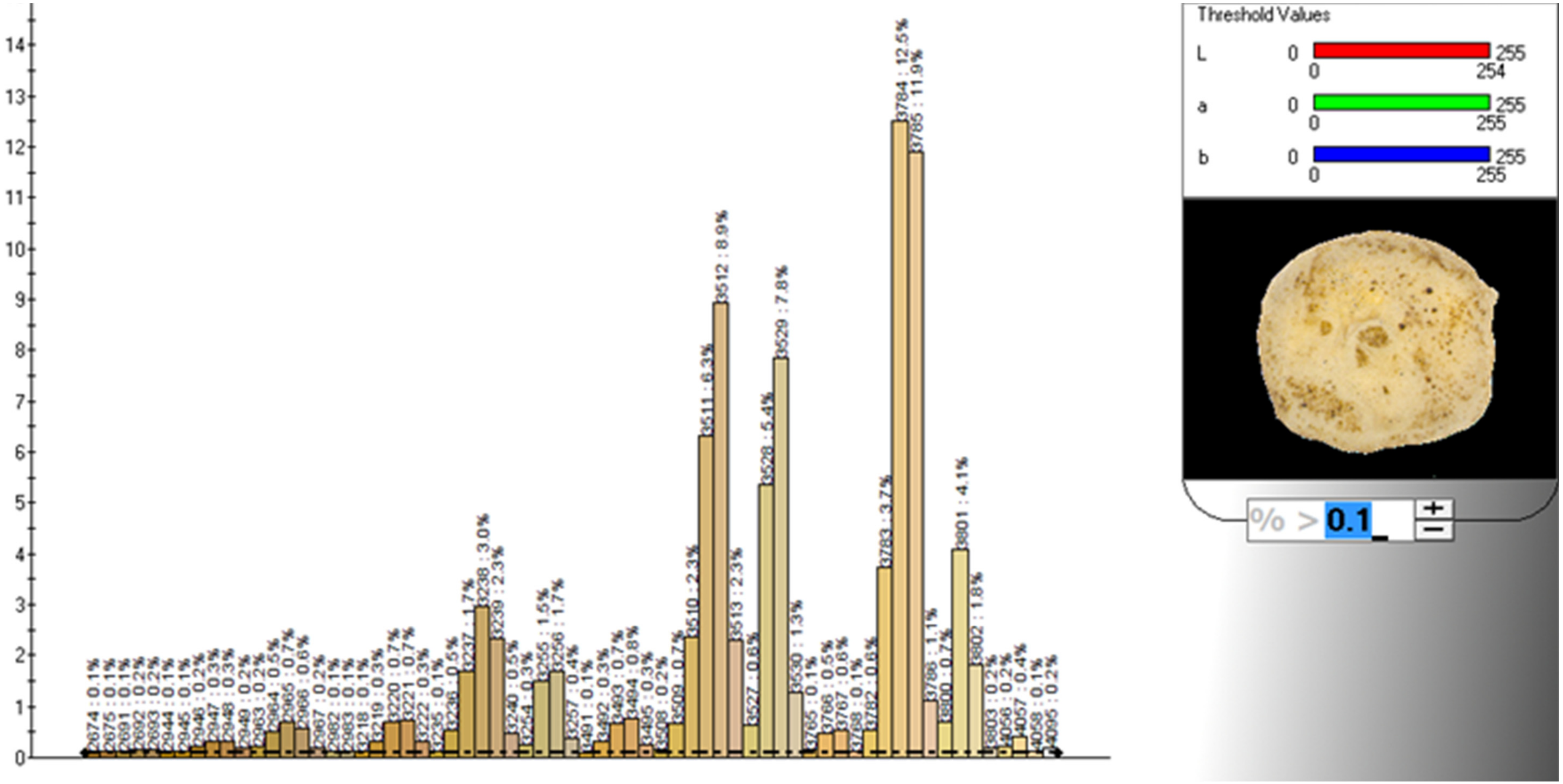

e) **Upper side of pizza sample C**

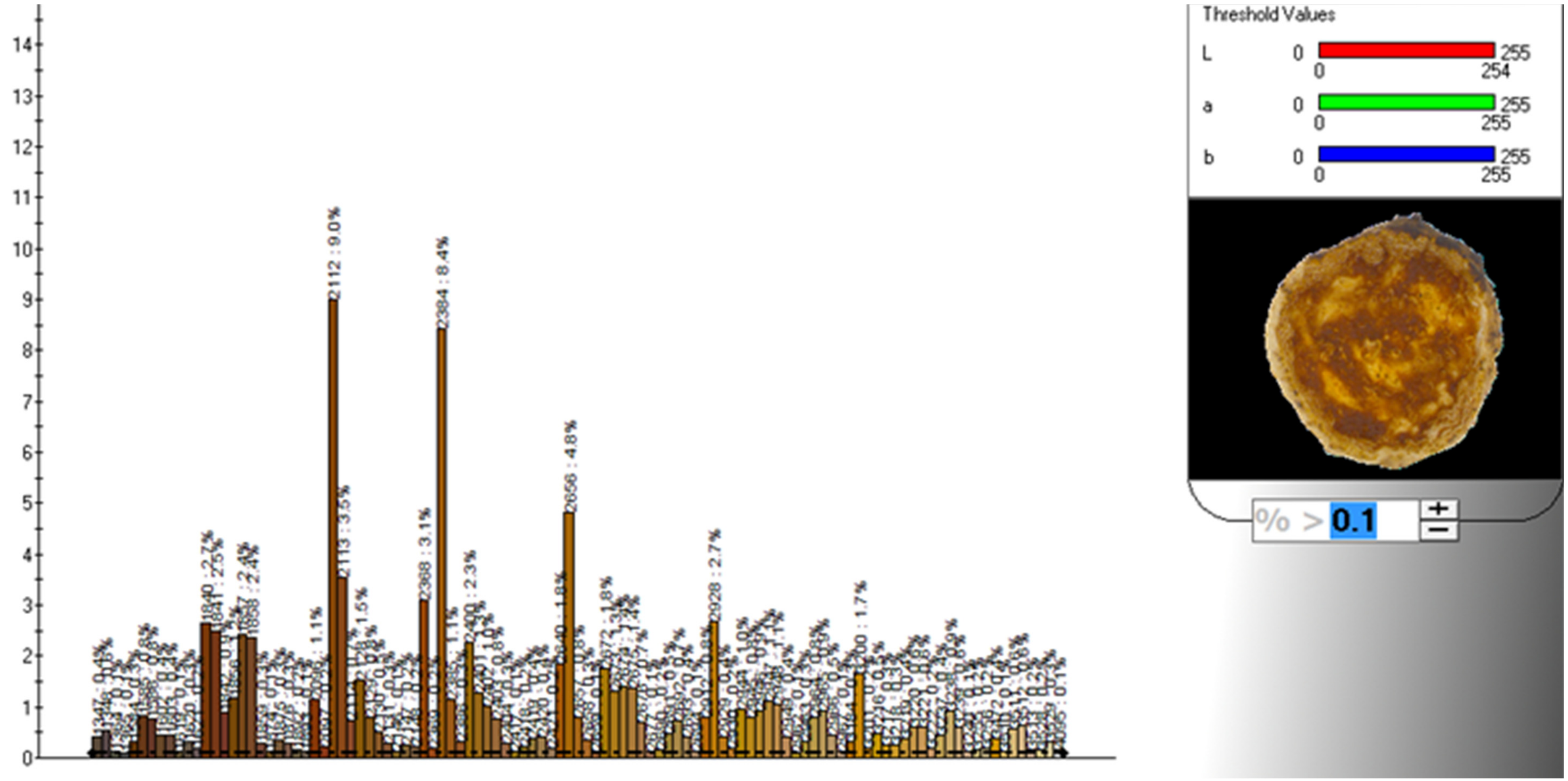

f) Lower side of pizza sample C

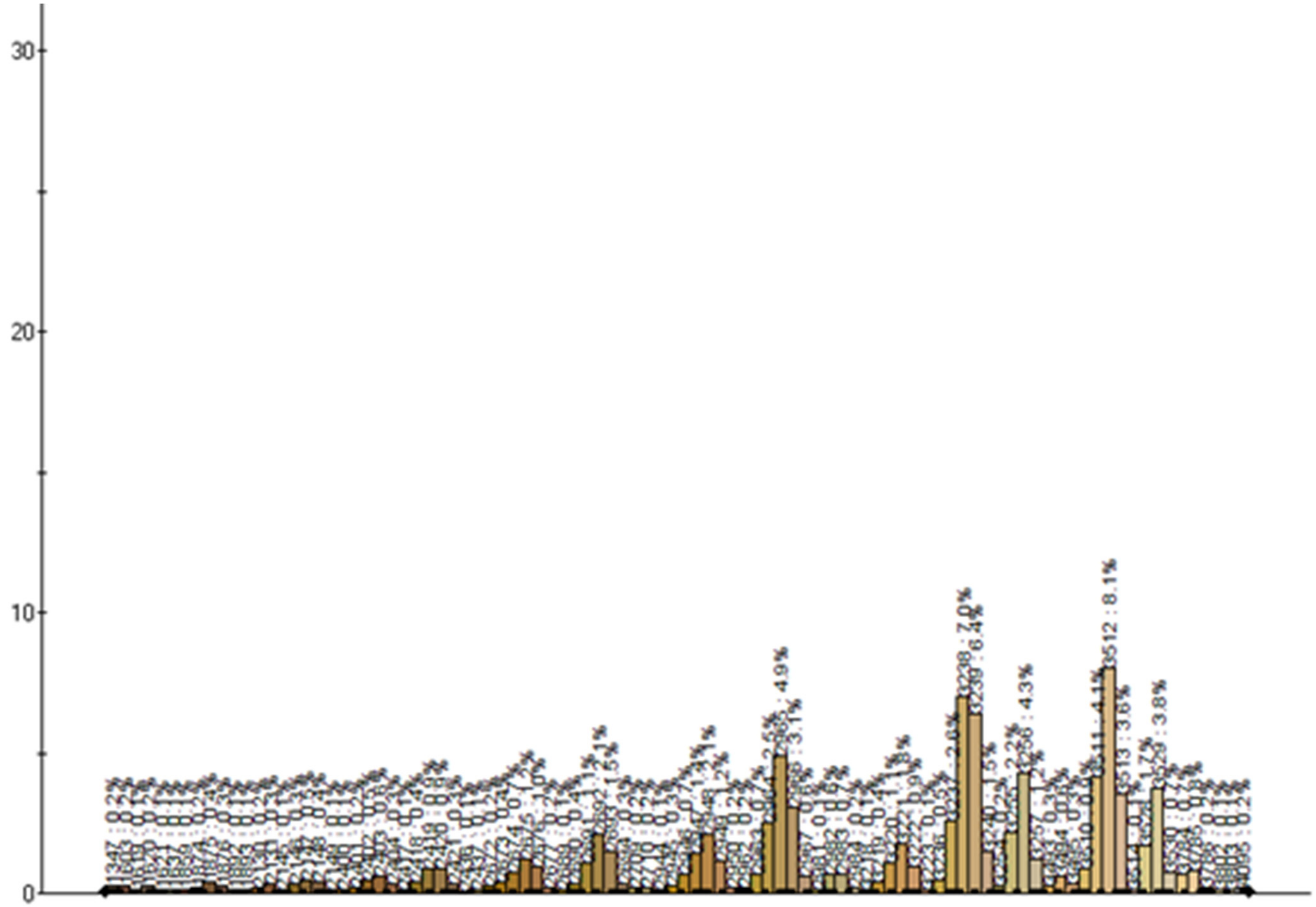

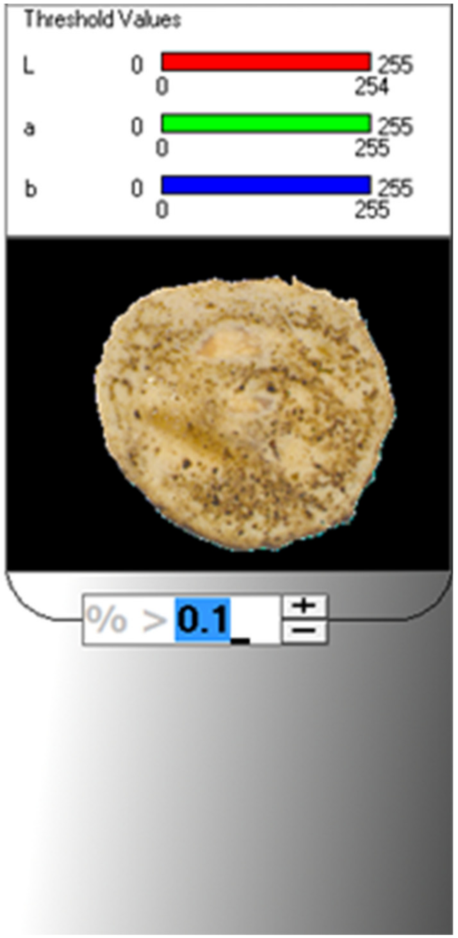

g) Upper side of pizza sample D

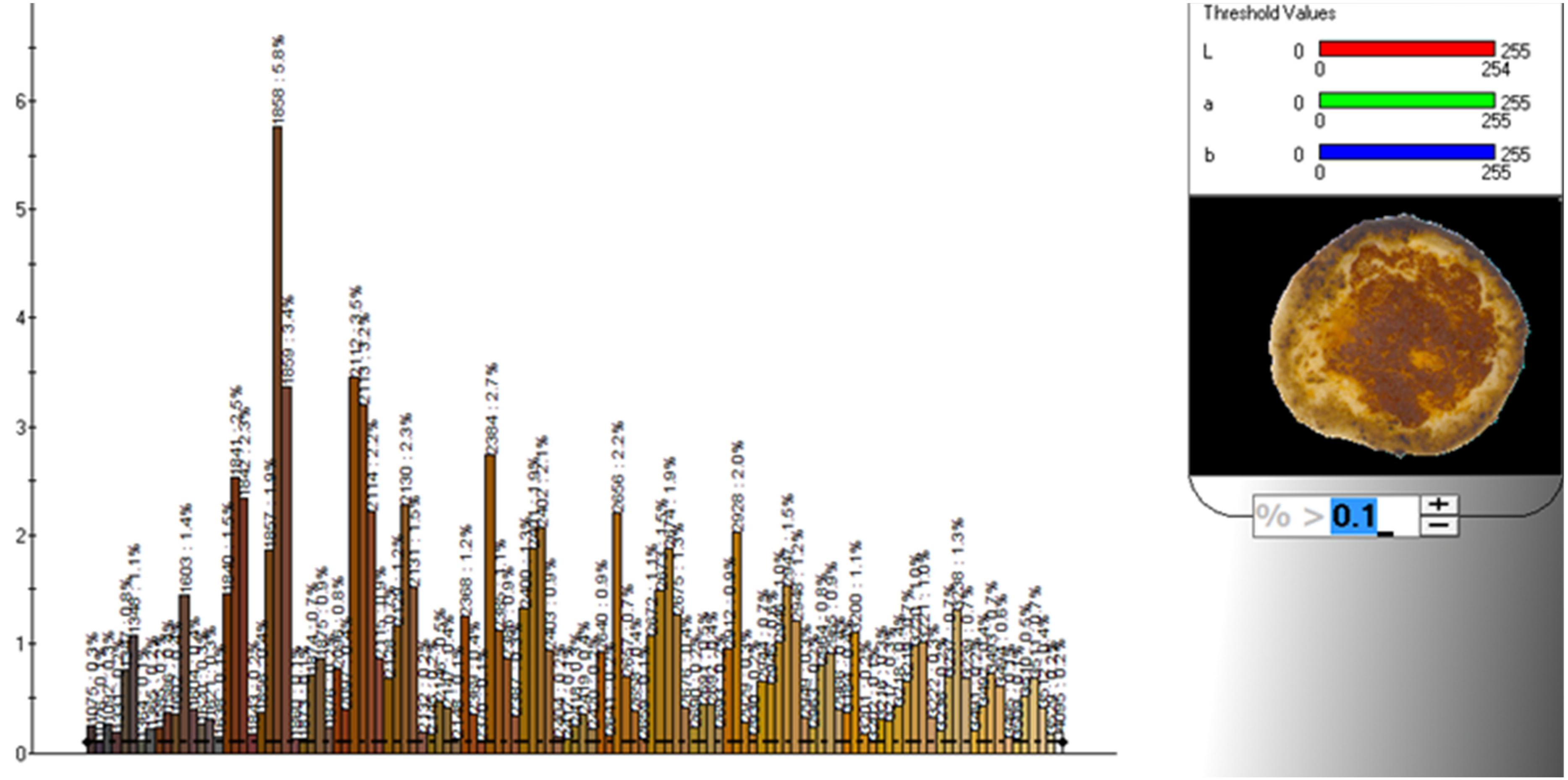

h) Lower side of pizza sample D

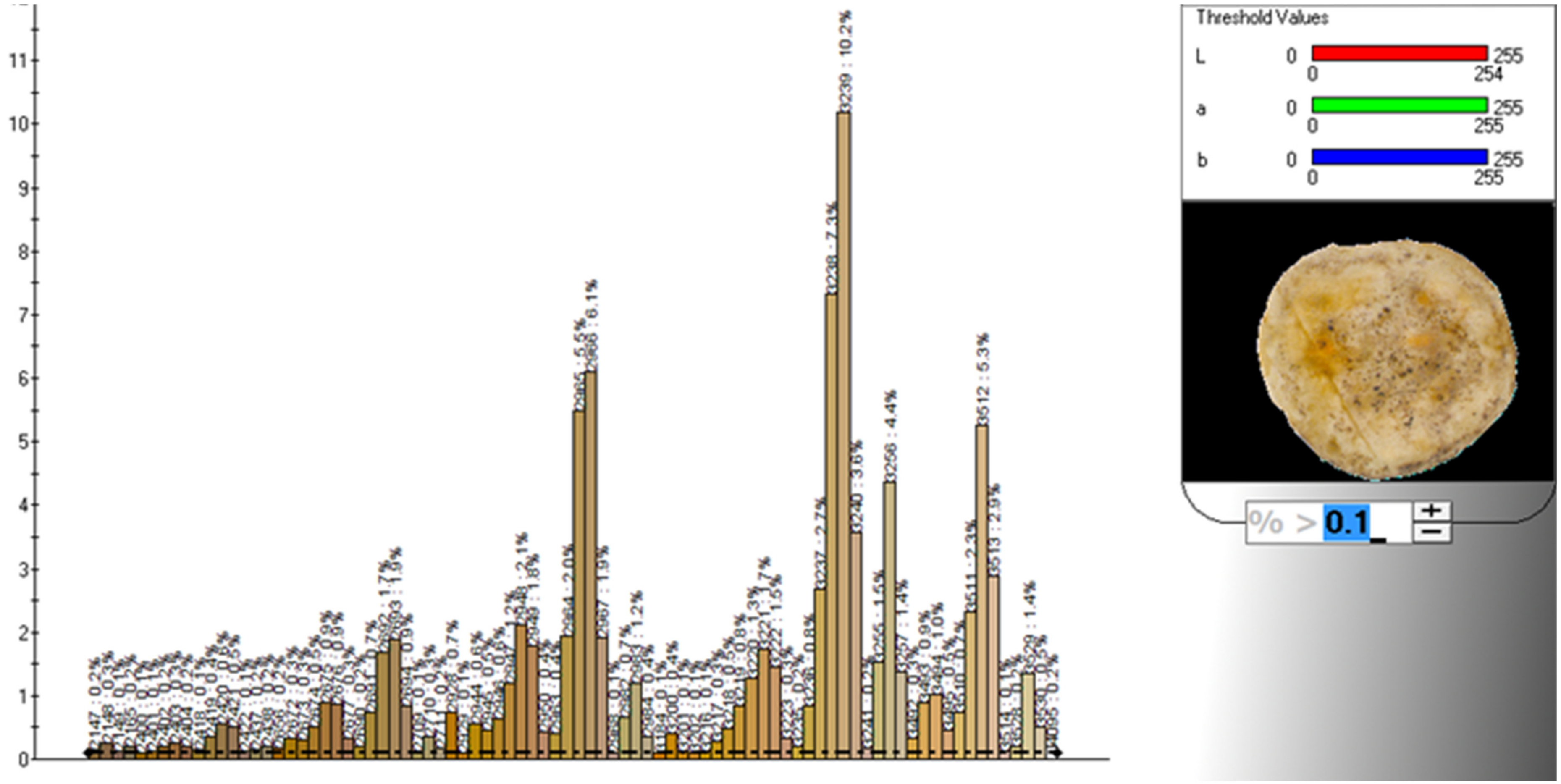

i) Upper side of pizza sample E

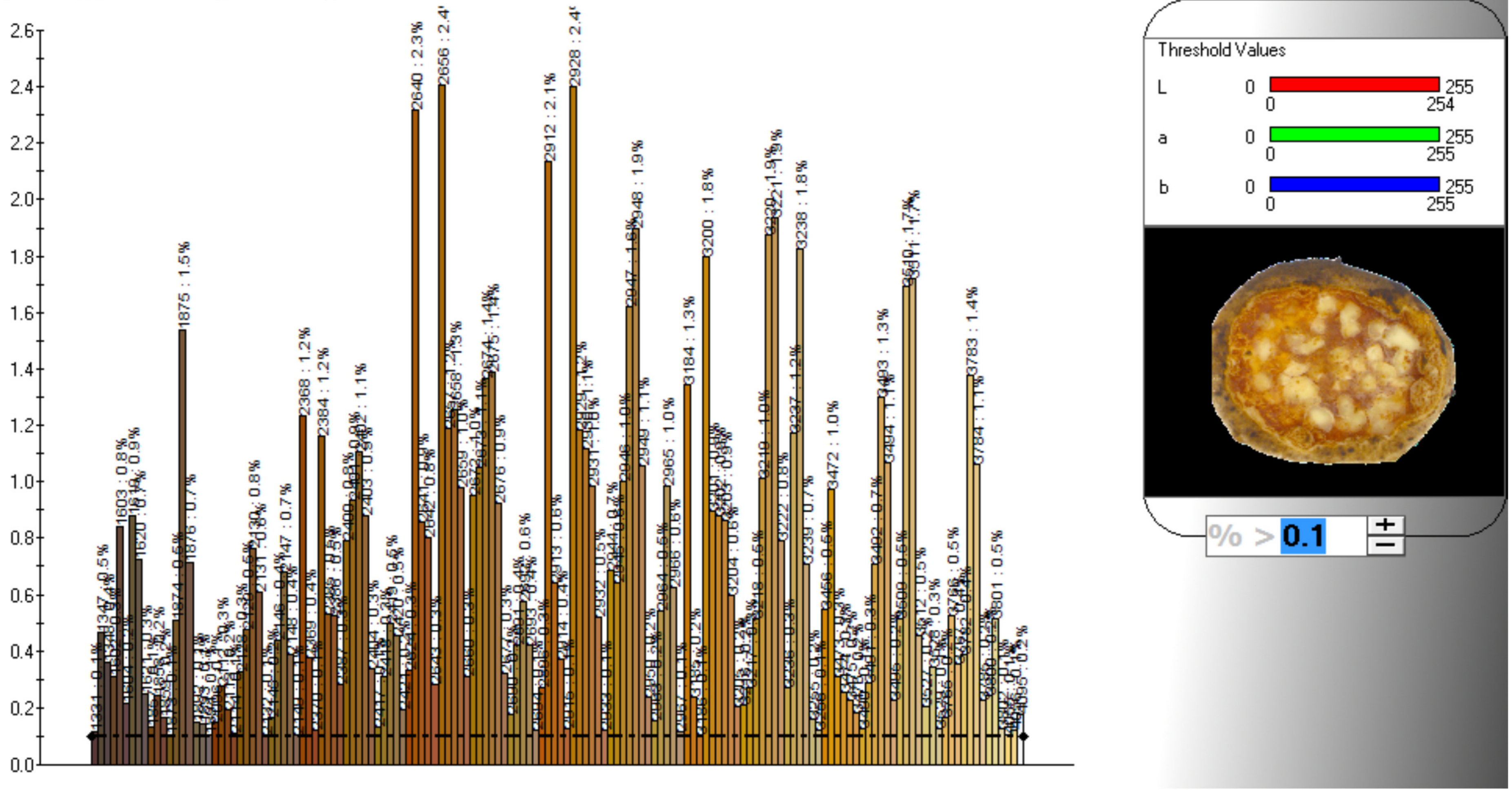

j) Lower side of pizza sample E

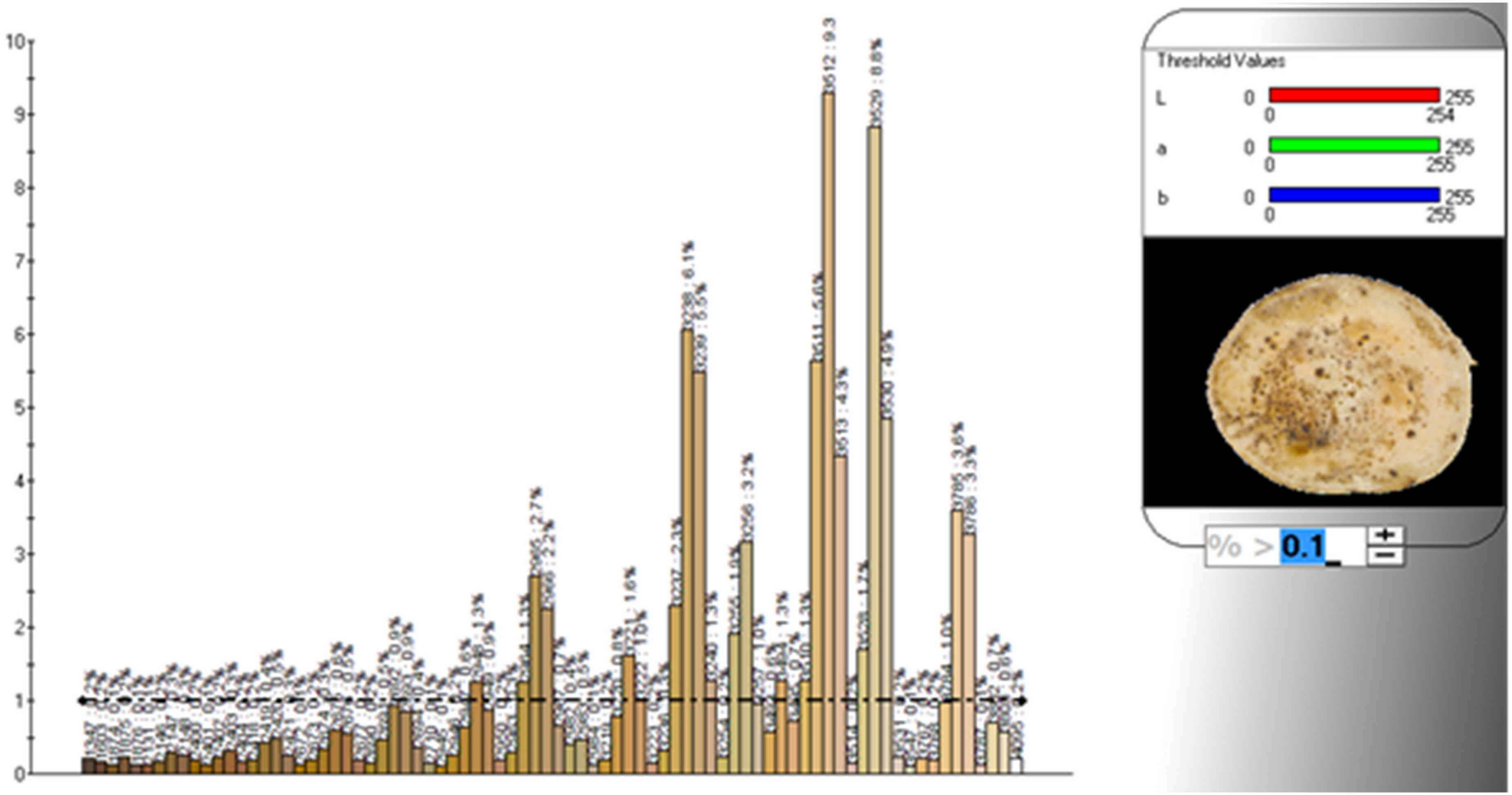

Supplement: Supplementary file 1 [file foods-12-00890-s001.zip › foods-2215228-supplementary.pdf]
